# Hormone and implant osseointegration: Elaboration of the relationship among function, preclinical, and clinical practice

**DOI:** 10.3389/fmolb.2022.965753

**Published:** 2022-09-15

**Authors:** Ming Yi, Ying Yin, Jiwei Sun, Zeying Wang, Qingming Tang, Cheng Yang

**Affiliations:** ^1^ Department of Stomatology, Union Hospital, Tongji Medical College, Huazhong University of Science and Technology, Wuhan, China; ^2^ School of Stomatology, Tongji Medical College, Huazhong University of Science and Technology, Wuhan, China; ^3^ Hubei Province Key Laboratory of Oral and Maxillofacial Development and Regeneration, Wuhan, China; ^4^ Department of Oral and Craniomaxillofacial Surgery, Shanghai Ninth People’s Hospital, Shanghai Jiao Tong University School of Medicine, Shanghai, China; ^5^ Shanghai Jiao Tong University School of Medicine, Shanghai Ninth People’s Hospital, Shanghai, China

**Keywords:** hormones, dental implant, osseointegration, PTH, insulin

## Abstract

As clusters of peptides or steroids capable of high-efficiency information transmission, hormones have been substantiated to coordinate metabolism, growth, development, and other physiological processes, especially in bone physiology and repair metabolism. In recent years, the application of hormones for implant osseointegration has become a research hotspot. Herein, we provide a comprehensive overview of the relevant reports on endogenous hormones and their corresponding supplementary preparations to explore the association between hormones and the prognosis of implants. We also discuss the effects and mechanisms of insulin, parathyroid hormone, melatonin, vitamin D, and growth hormone on osseointegration at the molecular and body levels to provide a foothold and guide future research on the systemic conditions that affect the implantation process and expand the relative contraindications of the implant, and the pre-and post-operative precautions. This review shows that systemic hormones can regulate the osseointegration of oral implants through endogenous or exogenous drug-delivery methods.

## Introduction

Tooth loss is one of the most common oral diseases in the population that seriously affects the quality of life. A meta-analysis published in 2010 pointed out that, although the number of cases has declined when compared with the previous 10 years, the incidence remained high after stratifying by age. In this regard, the worldwide age-standardized incidence rate of severe edentulousness was up to 205 cases per 100,000 person–years (Kassebaum et al., 2014), with the recent systematic review combined with data found that the average annual loss of attachment was 0.1 mm/year (95% CI 0.068, 0.132; I 2 = 99 %), and the average annual edentulousness was 0.2 teeth per year (95 % CI 0.10), 0.33; I 2 = 94 % (Needleman et al., 2018).

The causes of tooth loss include tooth decay, periodontal disease, trauma, cancer, wear and tear, and so on, which can cause extra dental issues, nutritional changes, and personal self-esteem and social identity issues. Titanium implants are currently one of the most effective treatments for tooth loss. The 10-year survival rate is as high as 90–97 %. However, a more rigorous meta-analysis published in 2019 suggested that the implant survival rate was overestimated, especially in the elderly, where it was reportedly double (Howe et al., 2019). Increasing osseointegration may still be an important direction to increase the success rate of implants in the population. Osseointegration refers to the direct contact between a living bone and the implant’s surface and was first proposed by the Swedish scientist Per-Ingvar Brånemark and his coworkers. It was later called ‘functional ankylosis’, given that it formed the basis for the success of oral implants. Although the precise molecular mechanism of osseointegration remains unclear, it has been established that successful osseointegration in wound healing can roughly be divided into: 1) The hemostatic phase, where the platelets attach to the wound with the fibrinogen polymerization, lasting from a few minutes to a few hours. 2) The acute inflammation phase, featuring the migration of inflammatory cells and the release of inflammatory mediators, usually lasting from 10 minutes to a few days after surgery. 3) The proliferation phase, during which new extracellular matrix (ECM) and blood vessels are deposited, accompanied by the formation of new bone, which lasts from a few days to several weeks. 4) The remodeling phase, where bones are remodeled according to functional needs under the interaction of osteoclasts and osteoblasts; this process can last for several years. These four stages of osseointegration are not distinct but overlap with individual variations present (Terheyden et al., 2012).

Under physiological conditions, various systemic hormones play a non-negligible role in the metabolic processes of bone growth, repair, reconstruction, and aging. Hormone disorders (e.g., insulin, parathyroid hormone, etc.) are also positively related to skeletal diseases, such as osteoporosis, osteoarthritis, and acromegaly. Correspondingly, targeting these hormones can prevent, regulate, or even reverse the progression of bone-metabolic diseases to a certain extent (August et al., 2001; Cauley, 2015; Bassett and Williams, 2016; Lecka-Czernik, 2017; Compston, 2018a; Maffezzoni and Formenti, 2018; Li T. et al., 2019) (Table 1).

**TABLE 1:** Animal experiments and/or clinical case evidence related to implants and hormones in the past 2 decades.

Hormones	Author	Study design/Experimental model	Subjects and number	Results	Year
Thyroxine	Ursomanno et al. (2020)	Retrospective study	Patient N = 635	Patients with HT have a decreased rate of bone loss	2020
	Feitosa dda et al. (2008)	animal experiment	Wistar rat	Osseointegration ↑healing process in the cortical bone around titanium implants	2008
			N = 43		
Estrogen	August et al. (2001)	Retrospective study	Patient N = 526	Estrogen deficiency may be risk factors for maxillary implant failure	2001
	Pan et al. (2000)	animal experiment	female Wistar rats	The bone mass around the implant and the BIC in the cancellous bone area ↓	2000
			N = 18		
	Duarte et al. (2003)	animal experiment	female Wistar rats		2003
			N = 30		
Androgen	Maus et al. (2013)	animal experiment	SD rats N = 20	Dihydrotrophil 2 promotes cobalt-chromium implant bone bonding	2013
Growth hormone	Tresguerres et al. (2002)	animal experiment	New Zealand rabbits N = 8	(topical application) Periimplant bone reaction and mineralization of osteoid ↑	2002
	Stenport et al. (2001)	animal experiment	New Zealand rabbits N = 16	(Systematic administration) Improving Initial implant stability	2001
	Calvo-Guirado et al. (2011)	animal experiment	Beagle dogs	(Topical application), BIC is not significantly affected	2011
			N = 12		
Melatonin	Calvo-Guirado et al. (2010)	animal experiment	Beagle dogs	BIC↑	2010
			N = 12		
	Sun et al. (2020)	animal experiment	SD rats N = 30		2020
	Palin et al. (2018)	animal experiment	Wistar rat N = 18		2018
	Salomó-Coll et al. (2016b)	animal experiment	American Foxhound dogs = 5		
Glucocorticoid	Petsinis et al. (2017)	Retrospective study	Patient N = 31	Glucocorticoid intake did not affect BIC and 3-year survival of dental implants	2017
	Keller et al. (2004)	animal experiment	New Zealand rabbits N = 40	The resulting osteoporosis affects BIC	2006
Insulin	Ferreira et al. (2006)	Retrospective study	Patient N = 121	diabetes caused by abnormal insulin metabolism are more likely to cause peri-implantitis	2006
	Wang et al. (2011)	animal experiment	Wistar rat N = 30	Direct insulin infiltration improves BIC	2011
	de Molon et al. (2013)	animal experiment	Wistar rat N = 80	Insulin deficiency made BIC、torque value↓	2013
	Coelho et al. (2018)		Göttingen minipig N = 9		2018
	Rybaczek et al. (2015)	animal experiment	Wistar rat N = 40	no change in bone area around medullary implants or in bone and BIC	2015
Adiponectin	Yin et al. (2019)	animal experiment	Female SD rats	BIC↑	2019
			N = 18		
PTH	Kuchler et al. (2011a)	Clinical Randomized Controlled Trial	Patient	NBV/TV↑	2011
			N = 24		
	Daugaard et al. (2012)	animal experiment	American Hound dogs N = 20	osseointegration of cancellous bone↑	2012
	Gomes-Ferreira et al. (2020)	animal experiment	Wistar rat N = 24	Bone volume, mass and bone turnover↑	2020
	Park et al. (2020)	animal experiment	SD rats	Promoting bone formation around implants	2020
			N = 30		
Vitamin D	Guido Mangano et al. (2018)	Retrospective study	Patient N = 822	Vitamin D deficiency incidence of early implantation failure is increasing	2016
	Salomó-Coll et al. (2016a)	animal experiment	Foxhound dogs N = 6	Topical application of vitamin D to dental implants can reduce alveolar bone loss, BIC↓	2016
	Dvorak et al. (2012)	animal experiment	SD rats	Vitamin D deficiency has a negative effect on bone formation around cortical implants	2012
			N = 51		
IGF-I	Tang et al. (2015)	animal experiment	Mongrel dog N = 25	Implant bone repair after tooth extraction ↑	2000
CGRP	Yuan et al. (2020)	animal experiment	C57BL/6 or CGRP—/–	CGRP promotes macrophage polarization and enhances osseointegration	2020

**FIGURE 1:**
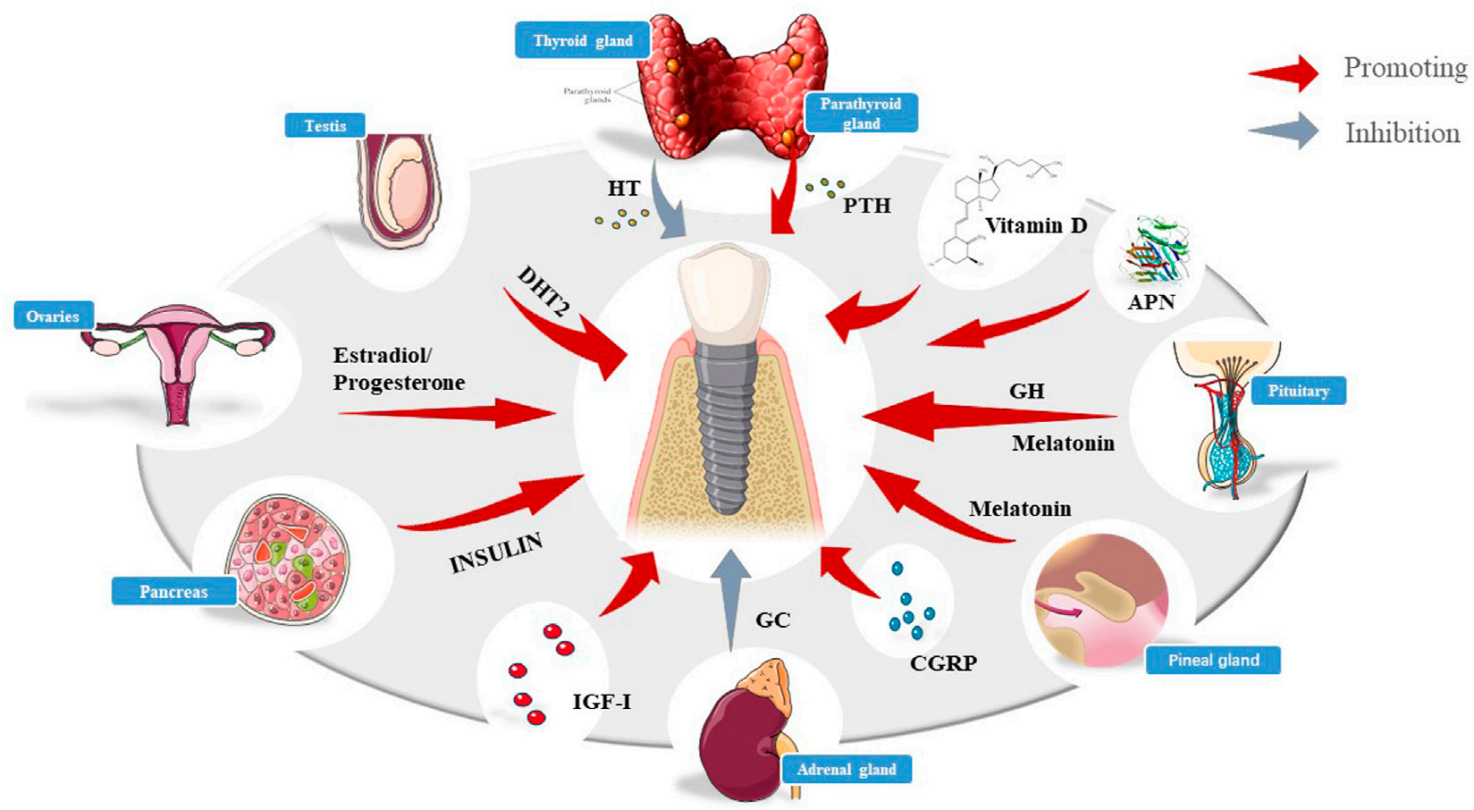
Schematic diagram of various hormones affecting implants, including insulin, HT, PTH, DHT2, melatonin, vitamin D, GC,IGF-Ⅰ,and so on.

It is widely acknowledged that patients with diabetes, older women, and other patients with hormone metabolism disorders are associated with a higher clinical failure rate, emphasizing the need to investigate the effect of systemic hormone metabolism on osseointegration. As mentioned earlier, osseointegration consists of four periods, among which the osseointegration proliferation period and the process of remodeling are the most critical periods since they directly determine the success of osseointegration. Skeletal anabolism and catabolism are dynamic processes. The remodeling of bone involves trade-offs and homeostasis between osteoblasts and osteoclasts. Hormonal disorders may disrupt the metabolism within these four phases, especially the imbalance between osteoblasts and osteoclasts, leading to structural and functional abnormalities in the bone which may ultimately culminate in implant failure. Few clinical and preclinical experiments have hitherto been reported where hormones have led to significant differences in the success rate of implants (Figure 1).

### The hyperglycemic conditions caused by the absolute or relative lack of insulin affects the implant osseointegration rate

#### Background

Secreted by pancreatic β cells, insulin is a unique hormone in the body that can decrease blood glucose levels. It is widely acknowledged that an absolute or relative deficiency caused by impaired insulin secretion or other factors can lead to diabetes (Kahn, 2003). Epidemiological studies suggest that the prevalence of diabetes has reached 5 % worldwide (Shaw et al., 2010). Diabetes can be divided into two categories. Type 1 diabetes (T1DM) is an autoimmune disease that usually occurs in children and accounts for 5–10 % of diabetes globally. Islet cell antibodies have been associated with the pathogenesis of T1DM, leading to the destruction of β cells. Accordingly, patients require exogenous insulin throughout their lives. Type 2 diabetes mellitus (T2DM) is the most common disease (more than 90 % of diabetic patients suffer from T2DM) and is reported to be highly hereditary, although other risk factors, including age, pregnancy, and obesity, have also been reported. It has been established that in T2DM, β cells secrete sufficient insulin to meet the required functions caused by type 2 diabetes mellitus (T2DM) (Rorsman and Braun, 2013). In short, the fact that the homeostasis of insulin and other glucocorticoid hormones is disrupted, resulting in the absolute or relative insufficiency of insulin will lead to diabetes.

Among all the complications of diabetes, adverse effects on bone metabolism affect people’s quality of life in all aspects, including oral implant osseointegration and peri-implant inflammation (de Oliveira et al., 2020). Hyperglycemia usually leads to microvascular and macrovascular diseases and poor wound healing, including the generation of advanced glycation end products (AGEs) and the increase of their receptors (receptor for advanced glycation end products, RAGEs) that induce the formation of reactive oxygen species (ROS), inflammatory cytokines, and the activation of protein kinase C isoforms, eventually leading to vascular damage (Stratton et al., 2000). Moreover, Das et al. proposed that diabetes mellitus-induced long noncoding RNA dnm3os can upregulate the macrophage inflammatory phenotype via nuclear mechanisms (M1) (Das et al., 2018), which has a negative impact on osseointegration (Wang et al., 2018). Most importantly, hyperglycemia exerts a regulatory effect on osteoblasts and osteoclasts; chronic hyperglycemia reduces the ratio of bone biochemical markers, including osteocalcin, AKP, and procollagen type 1 N terminal propeptide, runt-related transcription factor (RUNX2) (Compston, 2018b). In addition, the microenvironment of increased plasma glucose generated by insulin disorders can trigger the oxidative stress of osteoblasts and the formation of osteoclasts mediated by the Receptor Activator for Nuclear Factor-κB Ligand (RANKL) (Rathinavelu et al., 2018). Therefore, under hyperglycemic conditions, osseointegration and its maintenance may be impaired with the imbalance of these critical factors.

### Clinical cases and animal experiments

Uncontrollable diabetes has consistently been one of the relative contraindications of implants. Numerous clinical prospective and retrospective experiments have also alarmed that, compared with the general population, the bone-healing response after implant placement is impaired, and wound healing is delayed in diabetic patients with uncontrolled blood sugar levels, resulting in a higher implant failure rate, most of which occur in the first year of functional loading, with an increased risk of peri-implantitis (Annibali et al., 2016). In the current clinical studies, in addition to conventional indicators, hemodynamics model assessment (HOMA) was used to quantify insulin resistance (HOMA-IR), HOMA-IR = fasting blood glucose (FBG)× fasting insulin (FINS)/22.5; beta-cell function (HOMA-β), HOMA-β = 20×(FINS/FBG-3.5), and insulin sensitivity (HOMA-IS) HOMA-IS = 1/HOMA-IR, the unit of fasting blood glucose is mmol/L, with μU/mL in fasting insulin, which gradually become the references of short- or long-term clinical cases (Yary et al., 2016). In a 7-year follow-up, compared to non-diabetic patients, patients with type 2 diabetes experienced significantly more average peri-implant bone loss (Al Zahrani and Al Mutairi, 2018), while the implant success rate in patients treated with insulin and other interventions and controlled blood sugar levels was comparable to that of ordinary people. Moreover, the levels of osteopontin, receptor activator of nuclear factor-kB ligand (RANKL), and IL-8 were significantly higher than in diabetic patients (Sundar et al., 2019; Sghaireen et al., 2020).

**FIGURE 2:**
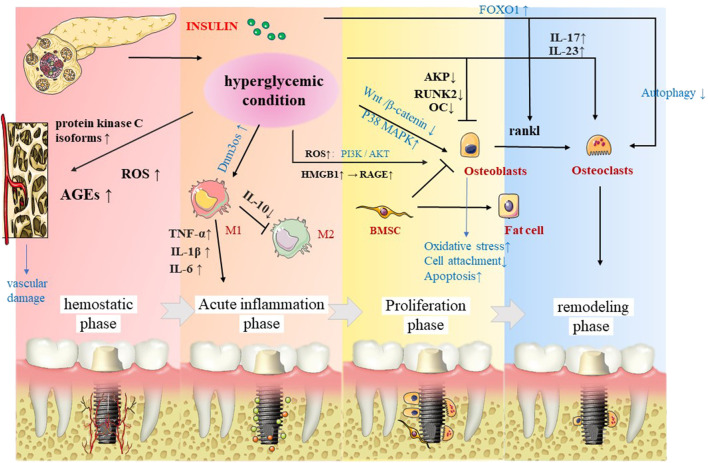
In the four stages of osseointegration, insulin significantly affects the implants not only directly by osteoblasts and osteoclasts, but also affects the implants through macrophage polarization, as well as vascularization.

The aforementioned findings were consistently found in preclinical experiments. In this regard, a study found that the osseointegration of diabetic animals was significantly lower than that in the control group, and the serum osteocalcin level of diabetic animals was increased, while alkaline phosphatase decreased 14 days after implantation (McCracken et al., 2000). An increasing amount of evidence suggests that diabetes leads to a significant reduction in bone formation and bone–implant contact in the cortex and bone marrow areas of Wistar rats, the mRNA levels of TNF-α, IL-1β, RAGE, and AGEs are elevated, and the lowest torque values are associated with implant removal (Kwon et al., 2005; Hasegawa et al., 2008; de Molon et al., 2013; Yamazaki et al., 2020). Furthermore, a study found that the mineral apposition rate was significantly reduced after immediate implantation in the oral cavity of diabetic rats (Shyng et al., 2006). Paulo G Coelho et al. found that diabetes mellitus (DM) piglets showed bone-healing disorders, while no significant alterations in bone formation capacity were observed (Coelho et al., 2018). Similar findings were found in another study (Schlegel et al., 2013), suggesting the negative impact of untreated diabetes in the early osseointegration of dental implants. After insulin treatment, the ultrastructural characteristics and osseointegration rate of the bone–implant interface are reportedly improved (Kwon et al., 2005), suggesting that controlling hyperglycemia by hormone control during dental implant surgery is a key strategy to achieving higher survival rates (Figure 2).

#### Molecular mechanism

It is well-established that insulin activates multiple cascades of intracellular signaling pathways, characterized by phosphorylation events and then inactivates the forkhead box protein (FOX). Under stress conditions, insulin and growth hormone levels decrease, and FOXOs are activated and mediate the osteocyte function, among which, FOXO1 can mediate the generation of osteoclasts by regulating RANKL. At the same time, the hyperglycemic environment may weaken the autophagy of osteoclasts and increase the production of osteoclasts. For mesenchymal stem cells (MSC), a hyperglycemic microenvironment can promote the production of fat cells and inhibit osteoblasts, which eventually cause bone loss (Botolin et al., 2005). Overwhelming evidence substantiates that DM can induce hyperphosphorylation of P38 MAPK in osteoblasts on titanium surfaces but does not induce hyperphosphorylation of ERK or JNK, indicating that the P38 MAPK pathway is a candidate ROS-sensitive signaling pathway, inciting impaired bone formation (Wang et al., 2016). Moreover, the Wnt/β-catenin pathway promotes the differentiation of progenitor stem cells into osteoblasts and prevents adipogenesis. However, experiments have shown that this pathway is inhibited in osteoblasts on titanium surfaces and prevents cell attachment and functions through osseointegration (Ma et al., 2014). Diabetes-related osteoporosis is partly due to the formation and accumulation of advanced glycation end products, which can also inhibit the adhesion of osteoblasts to the titanium surface (Fiorellini et al., 2020). Diabetes also reportedly hampers osseointegration by delaying the expression of fibronectin and integrin α5β1 (Liu et al., 2015).

It is well known that oxidative stress (OS) plays a key role in the pathogenesis and complications of aging and aging-related metabolic diseases such as osteoporosis, osteoarthritis, and diabetes and impairs the process of osteogenesis. Several metabolic pathways stimulate OS development under hyperglycemic conditions, including the glycolytic pathway, advanced glycation end product (AGE) formation, hexosamine pathway, activation of protein kinase C (PKC), and inactivation of the polyol pathway and the insulin-signaling pathway. Current evidence suggests that Sirtuin3 (SIRT3) is mainly located in the mitochondria and strongly impacts mitochondrial homeostasis, oxidative stress, and immune cell function. Interestingly, SIRT3 is reportedly reduced in DM, promoting mitochondrial oxidative stress and delayed fracture healing (Huang et al., 2022). Under diabetic conditions, reactive oxygen species (ROS) induce the phosphorylation of osteoblasts through the PI3K/AKT pathway, causing their dysfunction and apoptosis, and can affect osseointegration and implant failure through oxidative stress (Li et al., 2015; Saito et al., 2022). It has recently been shown that the high mobility group box 1 (HMGB1), a pathological cytokine, is related to diabetic osseointegration disorders and actively participates in oxidative stress. Moreover, RAGE is upregulated, inciting BMSC function damage, leading to obstacles for osseointegration (Liu et al., 2019).

Current evidence suggests that type 2 diabetes (T2DM) is an inflammatory disease, obesity is a major risk factor for T2DM, and adipose tissue is the main site of inflammation. Leukocyte trafficking dysregulation has been observed in adipose tissue and is associated with a shift in cell populations from being anti-inflammatory to pro-inflammatory in obesity. Shifting to the production of pro-inflammatory cytokines leads to low-grade systemic inflammation leading to impaired insulin signaling, beta-cell dysfunction, and subsequent insulin resistance (Pezhman et al., 2021). In young diabetic mice with periodontal disease, a shift in hyperglycemia has been shown to cause changes in the periodontal status, periodontal inflammation, and macrophage senescence. At the same time, GLUT1, as a typical glucose transporter, plays an important role in it. The expressions of both GLUT1 and the downstream GTPase Rheb are reportedly upregulated in the gingiva of diabetic mice, and mTOR phosphorylation in bone marrow-derived macrophage (BMDM) triggers SASP release and p16/p21 signaling, limiting glucose uptake by GLUT1 to antagonize −/−BMDM (Wang et al., 2021).

Moreover, its direct impact on bone-related cells, the DM environment indirectly impairs osseointegration by affecting blood vessels (Hu et al., 2018). Likewise, the role of inflammatory factors cannot be ignored. It has been reported that pro-inflammatory factors IL-17 and IL-23 in T1DM patients are increased, which promote the destruction by RANKL., the formation of bone cells, people with both T2DM and periodontitis also significantly elevated the levels of TNF-α, IL-1β, and IL-6 (Jiao et al., 2015). Moreover, under the premise of diabetes, the noncoding RNA Dnm3os is upregulated, its interaction with nucleolin and epigenetic modifications on target genes is disrupted, and these modified genes promote the production of the inflammatory phenotype of diabetic macrophages (Das et al., 2018), which has a negative impact on the acute inflammatory phase of osseointegration (Wang et al., 2018).

#### Translation and application

The past decade has witnessed significant inroads achieved in dental implant treatment for diabetic patients. It has been established that insulin can reverse osteoblast disorders, and the combined use of other agents can accelerate this process. Resveratrol, metformin, and other related drugs have also been found to promote the osteogenesis and osseointegration of diabetic alveolar bone mesenchymal stem cells (Hua et al., 2020; Sun et al., 2021). Moreover, material modifications such as micro- and nano-topographically complex implant surfaces can promote early bone anchorage in diabetic patients (Ajami et al., 2016). Intriguingly, Xing Wang et al. invented a new delivery method of uniform-sized, insulin-loaded PLGA microspheres for stable insulin delivery (Wang X. et al., 2019).

### By regulating the calcium and phosphorus levels, the parathyroid hormone affects the implant osseointegration rate

#### Research background related to stomatology

The parathyroid hormone (PTH) secreted by the parathyroid glands regulates the calcium and phosphorus metabolisms, promoting osteoblast activity and accelerating bone transformation. PTH secretion exhibits an ultradian rhythm consisting of tonic and pulsatile components (70 % tonic, 30 % pulsatile) to regulate the metabolism of calcium and phosphorus based on their serum levels. The half-life of the parathyroid hormone in the human body is about 2–5 min. As the PHT binds to the receptor, adenylate cyclase or phospholipase C is initiated, activating protein kinase A (PKA) or the PKC cascade reaction. It is generally believed that these pathways account for the bone formation and resorption phenotype induced by PTH. However, it should be borne in mind that other pathways may also affect the differentiation of MSC into osteoblast cell lines induced by PTH (Wojda and Donahue, 2018).

Both animal and human models have validated that the continuous increase of serum PTH (1–34) can induce catabolism, while an indirect increase leads to anabolism. Although the osteogenesis effects are similar, continuous administration can cause increased calcification and, ultimately, an increase in the trabecular bone (Tam et al., 1982). Another concept is that continuous versus intermittent PTH administration may induce bone turnover at the cortical and trabecular sections. It has been shown that continuous administration can result in osteoclast excitation and increased cellular span, potentiating endosteal resorption. In contrast, intermittent administration results in increased trabecular bone volume. Intermittent PTH treatment plays a major role in mediating the anabolism of osteoblasts. The main receptor of PTH and PTHrP is the G protein-coupled receptor PTH1R, which is well-recognized to be present on the membranes of osteoblasts, bone cells, stromal cells, T cells, and macrophages. As one of the main drugs broadly approved for osteoporosis therapy, recombinant human PTH(1–34) is used to treat osteoporosis by suppressing bone resorption and promoting bone formation, which reduces the risk of bone fractures (Kaback et al., 2008). One study documented a case of osteoporosis where osteointegration improved significantly after teriparatide (rh [1–34] PTH) treatment in a case of hemiarthroplasty accompanied by signs of aseptic loosening (Oteo-Álvaro et al., 2011).

As the main systemic mediator of phosphate and calcium homeostases in bones, PTH has attracted significant concern in recent years in light of its role in oral health and teeth (Oteo-Álvaro et al., 2011). It is widely thought that by increasing the number of osteoblasts, inhibiting their apoptosis, and reactivating static lining cells, PTH can promote bone formation. Moreover, PTH can indirectly stimulate osteoblast differentiation with the help of insulin-like growth factor 1. Furthermore, PTH produces ODF (also known as the receptor activator of nuclear factor kappa B ligand) and the cytokine osteoprotegerin (OPG). There is ample evidence suggesting that PTH positively affects the formation and reduction of the alveolar bone to stimulate osteoclasts to promote bone resorption (Aggarwal and Zavras, 2012). Another study reported that, oral PTH could promote the healing of the alveolar socket (Kuroshima et al., 2013). Given the ability of PTH to improve the bone conversion efficiency, preliminary experiments in rats involving orthodontic treatment have shown that, although PTH cannot promote tooth movement, it can reduce the recurrence rate of osteoporosis (Lee et al., 2018). The topical use of PTH/PTHrP in controlled-release hydrogels is also believed to mediate periodontal bone remodeling and improve orthodontic efficiency, reflecting the potential of PTH in orthodontic treatment. An experiment suggested that, using PTH could affect the eruption of rat teeth (Silva et al., 2016). Dental pulp stem cells (DPSCs) have been extensively studied in bone and tooth regenerations. It has been shown that the parathyroid hormone enhances the osteo/odontogenic differentiation of dental pulp stem cells (DPSCs) and stem cells from the apical papilla (SCAPs) via the P38 MAPK pathways (Ge et al., 2020; Pang et al., 2020). At the same time, more emphasis should be placed on the impact of PTH on the periodontal tissue (Silva et al., 2016; Li et al., 2017). Xu et al. proposed that the cementogenesis-enhancing effect of PTH on cementoblasts depended on the interaction of the PKA and p-ERK1/2 pathways (Xu et al., 2019). It has also been suggested that intermittent PTH promotes cementogenesis through ephrinB2-EPHB4 forward signaling (Li et al., 2021). Consistently, the indirect use of PTH can inhibit the infiltration of neutrophils to inhibit the inflammation of the apex, thereby preventing the damage to the root apex of the tooth (Otawa et al., 2015). An RCT showed that, within 72 h after implant surgery, 40 patients exhibited an earlier peak thyroxine loading compared with no prosthesis placement (Prati et al., 2013). Piotr Hadrowicz et al. advocated that the serum level of the parathyroid hormone is an effective indicator for predicting the bone condition around dental implants (Hadrowicz et al., 2017), by comparing the new bone-volume-per-tissue-volume (NBV/TV) and new bone-to-implant-contact (NBIC) parameters. U Kuchler et al.'s clinically controlled experiment in patients who had two implants in the mandible revealed that the NBV/TV of the experimental group and control was 15.4 vs. 17.6 % (11.3 vs. 16.5 % and 7.3 vs. 12.0 % in the cortical and medullary compartments, respectively) after receiving 20 µg teriparatide once a day for 28 days. These results provide the basis for Parritt’s fetal treatment after implantation (Kuchler et al., 2011a).

#### Animal experiments

PTH has been extensively used for implant osseointegration in experiments in rats, rabbits, dogs, and other experimental animals, yielding positive results. According to micro CT and biomechanical and tissue morphological analyses, the experimental group’s tensile strength or the resistance to reverse torque, bone-to-implant contact (BIC), BV/TV, trabecular thickness (Tb.Th), and trabecular number (Tb.N) were significantly improved compared with those of the control group, fundamentally increasing the quality and initial stability of cancellous bone osseointegration (Shirota et al., 2003; Corsini et al., 2008; Valderrama et al., 2010). However, other trials showed that, in the case of hyperglycemia, intermittent PTH does not affect the BIC area (16 ± 12 % to 16 ± 8 %; *p* > 0.05), and no obvious changes were noticed in the cortical compartments of all groups (Kuchler et al., 2011b). This finding provides a basis for clinically improving the success rate of implant surgery in a poor musculoskeletal environment. PTH supplementation can even reduce the effects of poor bone healing and low bone density in a smoking environment (Lima et al., 2013). It is widely believed that the extent to which PTH plays a role is also related to age. The effect of maturity is better on young people, and the effect on older people is also obvious (Mair et al., 2009; Jiang et al., 2018). In terms of dosage, the systemic administration of PTH (1–34) can still stimulate bone formation around titanium implants, even at low doses. At the same time, attempts have been made to combine PTH and other drugs to improve the osseointegration rate of implants (Li et al., 2018). It is worth noting that PTH can promote allogeneic bone and autologous bone grafting (Sheyn et al., 2013).

#### Molecular mechanism

**FIGURE 3:**
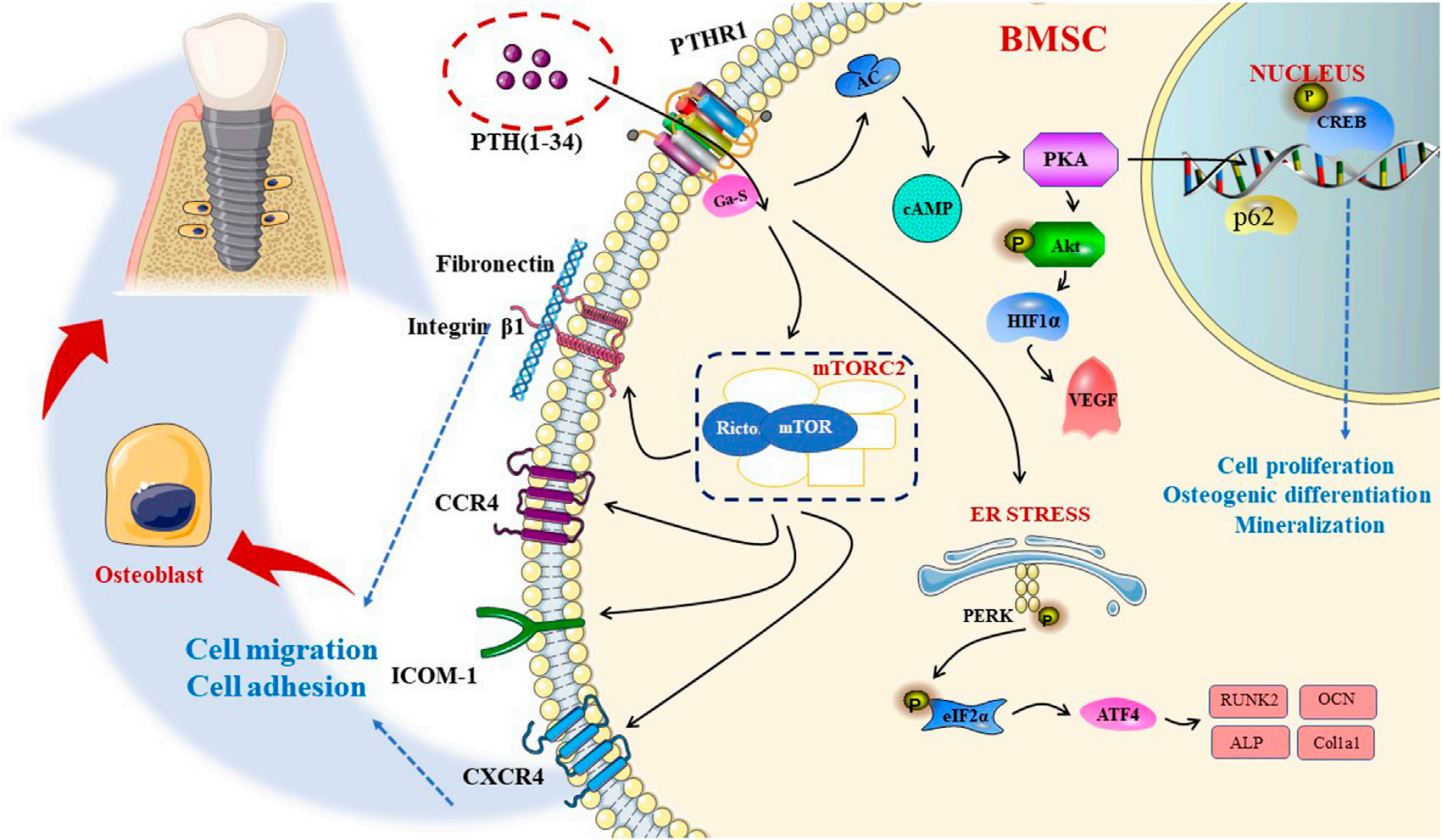
PTH affects the proliferation, differentiation, and migration of osteoblasts, respectively, through different pathways, including cAMP-PKA, mTORC2 pathways and so on, along with the ER stress to promote osseointegration.

mTOR is a non-classical serine/threonine protein kinase belonging to the phosphatidylinositol kinase-related kinase (PIKK) protein family. There are two diverse mTOR complexes in mammalian cells: mTORC1 and mTORC2. Lv et al. revealed that PTH (1–34) promotes BMSC migration and adhesion is dependent on rictor/mTORC2 signal transduction, suggesting that PTH can increase the protein expression of chemokine receptors (CXCR4 and CCR2) and adhesion factors [ICAM-1, fibronectin, and integrin β1], which substantiates the significant therapeutic effect of PTH(1–34) on osseointegration (Lv et al., 2020). In a study by Chen et al., intermittent PTH (1–34) administration could regulate downstream proteins in the cAMP/PKA signaling pathway, especially p-CREB, to stimulate the proliferation, osteogenic differentiation, and mineralization of BMSCs (Chen et al., 2016). Findings reported by Zhang et al. showed that PTH could induce osteoblast differentiation and proliferation, partly through the facilitation of the HSP90-dependent PERK-EIF2α-ATF4 pathway (Zhang et al., 2019). Recent studies by D. Agas et al. emphasized the indelible effect of p62. Compared with WT, p62-deficient mice could not increase their bone transformation rate via PTH, and the RUNX2 protein expression decreased, which substantiates the strong effect of the p62 gene on the bones in the presence of PTH (Agas et al., 2020). The scarcity of endogenous PTH may lower the expression of VEGF in osteoblasts by downregulating the PKA/pAKT/HIF1α/VEGF pathway, thus affecting endochondral skeleton formation by causing a decrease in angiogenesis compared to osteogenesis, resulting in delayed healing (Ding et al., 2018). In aging rats with implants, intermittent PTH (1–34) treatment could directly improve the ability of blood vessel formation and the osteogenic potential of aging BMSCs. Furthermore, PTH (1–34) triggers more osteoclasts to participate in bone remodeling by secreting angiogenesis and osteogenic growth factors; accordingly, it can induce early vascularization and indirectly stimulate the migration or differentiation of BMSCs (Jiang et al., 2018). During the process of fracture recovery, endogenous PTH deficiency can reduce the expression of RANKL in osteoblasts to indirectly inhibit osteoclasts, mainly mediated by the classical Wnt/β-catenin signaling, becoming indispensable for normal bone repair. This pathway not only mediates the differentiation of stem cells into osteoblasts, but also the proliferation, maturation, and inhibition of the apoptosis of osteoblast precursors (Figure 3) (Grosso et al., 2015).

#### Translation and application

In recent years, many breakthroughs have been achieved in combining materials and PTH. Xiaohua Yu et al. used coating technology to apply a CaP layer onto titanium implant surfaces as a carrier to complete the delivery of PTH local drugs, thereby increasing osseointegration (Yu et al., 2012). Jiahao Tang et al. deposited a hyaluronic acid/β-polylysine multilayer film containing PTHrP on a titanium implant layer by using layer electro-assembly technology for bone implantation. It was found that the osteoblast cell line MC3T3-E1 showed high levels of ALP activity and osteoblast-related protein expression *in vitro* (*p* < 0.05), and a significant initial increase in osteogenesis was observed *in vivo* (Tang et al., 2020). Zhou-Shan Tao et al. used another electrochemical deposition method to increase the strontium-doped hydroxyapatite coating (PTH + Sr-HA) for an intermittent local administration of the human parathyroid hormone (1–34); micro-CT and biomechanical evaluations were used to assess the improvement of osseointegration and to assist in confirming the feasibility of this method to improve dental osseointegration (Tao et al., 2016).

### Vitamin D deficiency: An easily overlooked factor that affects the implant osteointegration rate

#### Vitamin D and bone metabolism

Vitamin D is a fat-soluble biomolecule required by the human body, usually referred to as vitamin D2 (ergocalciferol) or vitamin D3 (cholecalciferol). Only cholecalciferol can be endogenously generated in the skin. The precursor of vitamin D3 is formed by the photolysis of 7-dehydrocholesterol (pro-vitamin D3) triggered by UV–B radiation from the skin. Vitamin D (D2 and D3) from the skin and diet undergo two sequential steps of hydroxylation: the first step is in the liver and the second one is in the kidney to obtain its biologically active form 1, 25-dihydroxyvitamin D (1.25 [OH] 2 D) (Holick, 2006). Importantly, Vitamin D actively participates in the formation, absorption, and mineralization of bones. Interestingly, 1.25 [OH] 2 D in the circulation inhibits serum PTH through negative feedback circulation and elevated serum calcium levels (Holick, 2004). Correspondingly, the increase in PTH increases calcium re-absorption by the renal tubules, promotes bone calcium mobilization, and enhances the production of 1.25 [OH] 2 D to restore calcium homeostasis (Holick, 2002). The small intestine can absorb about 10–15 % of dietary calcium at low vitamin D levels, leading to insufficient calcium absorption. Therefore, maintaining adequate levels of vitamin D is essential for normal metabolism, including bone health.

Vitamin D is a steroid hormone that significantly affects bone mineralization. A study found hypocalcemia, hypophosphatemia, and secondary hyperparathyroidism in mice with corresponding gene knockout (VDR−/− and (OH)ASE-/ -) given a normal diet, which was accompanied by skeletal morphological abnormalities, including insufficient mineralization of the cartilage, primary cavernous, and cortical bone, and increased non-mineralized osteoids in the cortical bone and trabecular bone (Panda et al., 2004). The function of vitamin D on skeleton formation, resorption, and turnover is achieved through the regulation of osteoblasts and osteoclasts (Rapuri et al., 2007). By affecting the expression of the growth factors interacting with other growth factors and hormones in the control of osteoblast differentiation, 1.25-(OH)2D3 regulates osteoblasts (Lian and Stein, 1993). Vitamin D sterol directly stimulates the maturation of osteoblasts (Pereira et al., 2019). *In vitro*, 1.25(OH)2 D is also an effective osteoclast stimulator. Large doses of 1.25(OH)2 D can combine with VDR in osteoblasts and increase the release of RANKL in osteoblasts, which increases the ratio of RANKL to OPG, increases the formation and role of osteoclasts, and affects bone resorption (Kitazawa et al., 2008). Indeed, vitamin D enables the regulation of the coupling of osteoblasts/osteoclasts/osteocytes, making it an extremely important part of implant osseointegration.

#### Related clinical reports

Case report studies have reported that serum vitamin D deficiency (Serum vitamin D level <20 μg/L) is related to the early failure of implant placement (Fretwurst et al., 2016). Retrospective clinical studies have shown that, although the incidence of early implantation failure is on the rise with the exacerbation of vitamin D deficiency, the association between low vitamin D content and early implantation failure is not statistically significant, which may be due to an insufficient amount of related cases (Mangano et al., 2016). Therefore, vitamin D is an important biological risk factor that is easily overlooked. A study was conducted on 90 implant sites in 53 included participants [healthy group around the implant (*n* = 30), mucositis around the implant (*n* = 30), and inflammation around the implant (*n* = 30)] and peri-implant sulcular fluid (PISF) samples were obtained in patients with different probe depth, clinical attachment level, suppuration, modified plaque index, gingival index, modified sulcus bleeding index, and keratinized mucosa width parameters. Analysis of the levels of 25(OH)D 3 by the enzyme-linked immunosorbent assay method found that the total amount of 25(OH)D 3 in the peri-implant inflammation group was significantly lower than in the healthy group (Choukroun et al., 2014).

#### *In vivo* and *In vitro* Experiments

In animal models, the lack of vitamin D caused unexpected serious damage to the osseointegration of titanium plants in the body. Compared with the control group, the BIC of Sprague–Dawley rats in the vitamin D deficiency group was significantly reduced after stopping the vitamin D intake and ultraviolet radiation. The SEM analysis showed that the calcified tissue remaining near the implant’s surface after the push-in test was abnormally fragmented (Kelly et al., 2009). In addition, the weighted gene co-expression network analysis of the SD rat implant femoral specimens showed that a large co-expression network containing Npas2, Bmal1, and Vdr was formed with titanium biomaterials, which was disintegrated by vitamin D deficiency. Therefore, it was pointed out that the circadian rhythm system and cartilage extracellular matrix may jointly participate in osseointegration under the regulation of vitamin D (Hassan et al., 2017). Systemic administration of vitamin D can significantly increase the percentage of BV/TV, Tb. Th, and Tb.N after implants in osteoporotic rats and mice with chronic kidney disease (Zhou et al., 2012; Liu et al., 2014). The combined use of vitamin D3 and insulin can also promote osseointegration of titanium implants in diabetic mice (Wu et al., 2013). In an experiment conducted by OSalomó-Coll et al., American Foxhound dogs were divided into: melatonin 5 % test group (MI), vitamin D 10 % test group (DI), and control implant (CI). After 3 months, block sections were obtained, and it was found that the topical use of 10 % vitamin D was also more effective in increasing the BIC and new bone formation (NBF) around the implant placed immediately after tooth extraction (Salomó-Coll et al., 2016a; Salomó-Coll et al., 2018), which substantiated that, in addition to the systemic use of vitamin D, local use around the implant can also help to form osseointegration and provide a variety of preparation possibilities for future administration.

Current evidence suggests that FoxO1 OB in osteoblasts may be involved in regulating glucose homeostasis and bone formation by 1.25 (OH) 2D3, thereby participating in the osseointegration of implants in diabetic mice (Xiong et al., 2017). The topical application of vitamin D around dental implants has been reported to improve alveolar bone loss and implant bone contact in American Foxhound dogs. (Salomó-Coll et al., 2016a). Olivares-Navarrete et al. proposed that 1.25(OH) 2D3 exerted a sex-dependent regulation of osteoblast differentiation on the morphology of the titanium substrate treated by acid etching and/or sandblasting (Olivares-Navarrete et al., 2015). In a recent prospective randomized controlled clinical trial, the experimental group was divided into three groups: Group A (*n* = 43) with 25-hydroxycholecalciferol in blood serum <30 ng/ml without vitamin D supplementation), group B (*n* = 48) with 25-hydroxycholecalciferol in blood serum <30 ng/ml, with vitamin D supplementation, and group C (*n* = 31) with normal levels of 25-hydroxycholecalciferol in blood serum (≥30 ng/ml). The results showed that after 12 weeks of the implant surgery, the bone 25-hydroxycholecalciferol levels around the implants in groups B and C were significantly higher than group A after 6 weeks, while levels in group B were significantly higher than in group A (Kwiatek et al., 2021).

#### Translation and application

The modification of vitamin D on the surface of implant materials is indispensable in promoting osseointegration. The 1α.25-dihydroxyvitamin d3 coating on the implant’s surface has made preliminary progress in achieving osseointegration in animal experiments (Naito et al., 2014). The biocompatible coating of titanium (Ti) implants of 7-dehydrocholesterol (7-DHC) (the precursor of vitamin D) activated by ultraviolet light can promote MSC and osteogenesis differentiation with varying degrees and durations of light, leading to a decreased Rankl gene expression (Naito et al., 2014). Attempts have been made to construct multilayered molecular reservoirs on implants for combinational drug delivery and 1α,25-dihydroxyvitamin D3-loaded hierarchical titanium scaffold. The former could upregulate the calcium-binding protein and BMP2 in osteoblasts, while the latter could promote early osseointegration (Chen et al., 2020; He et al., 2020).

### Both locally and systemically, melatonin can regulate the osseointegration rate

#### Melatonin and oral bone metabolism

Melatonin (N-acetyl-5-methoxytryptamine) is a neurohormone predominantly synthesized and secreted by the pineal gland in the brain (Reiter, 1991). Furthermore, it is also generated in other organs and tissues, including the spleen, thymus, ovary, testis, retina, intestine, and bone marrow. Only the melatonin secretion by the pineal gland and retina follows a classical circadian rhythm, whose secretion is inhibited by light and regulated by a circadian clock located in the hypothalamus. At the onset of darkness, the reduced retinal input leads to dis-inhibition of the enzymes responsible for melatonin synthesis. This increased nocturnal synthesis peaks in plasma concentrations between 2 and 4 a.m., with levels declining until the onset of the day, where low concentrations are observed (Lalanne et al., 2021). The past decade has witnessed significant scientific advancements, and melatonin has been found to participate in many biological activities in the body in recent years, such as the sleep–wake cycle, gastrointestinal physiology, immune defense, and bone physiology (Wang B. et al., 2019). The action of melatonin is accomplished by binding indoleamine to membrane receptors (MT 1, MT 2, and MT 3) or nuclear receptors (ROR/RZR) (Cutando et al., 2011).

The effect of melatonin on bone physiology is reflected in many aspects; the most direct is bone growth, bone remodeling, and bone repair (Liu et al., 2013). The main mechanisms of the effect of melatonin on bone metabolism include promoting the differentiation and activity of osteoblasts, inhibiting osteoclast differentiation, and scavenging free radicals to rescue osteoporosis (Luo et al., 2019).

**FIGURE 4:**
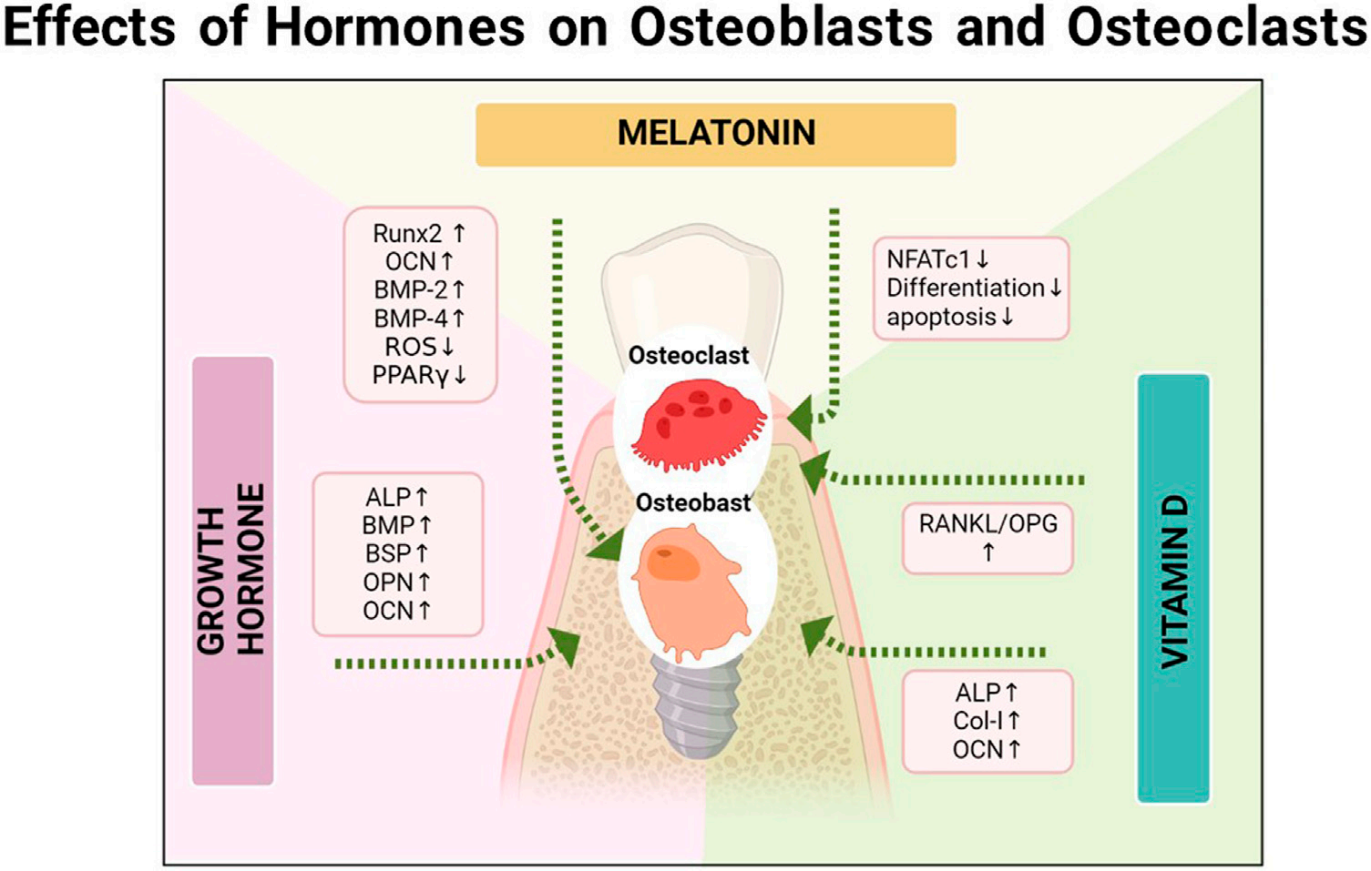
The relationship between the osseointegration and the vitamin D, melatonin, and growth hormone, respectively.

First, melatonin directly affects the role of osteoblasts and osteoclasts. At present, many studies have confirmed the regulatory capacity of melatonin on BMSC. It is well-recognized that melatonin’s osteogenesis is mainly mediated via MT2 receptors (Sharan et al., 2017). Moreover, melatonin can activate Wnt5α/β, β-catenin, phosphorylate JNK and ERK, and enhance cell differentiation, migration, and bone mineralization. At the mRNA level, Runx2, OCN, BMP-2, and BMP-4 are upregulated (Park et al., 2011). Furthermore, melatonin/BMP9 is believed to synergistically initiate the AMPKα/β-catenin pathway and contribute to efficient bone formation in BMSCs (Jiang et al., 2019). The PDGF/AKT signaling pathway also participates in osteoblast differentiation (Zhu et al., 2020). Under hypoxic conditions, osteoblastic differentiation and bone-forming capacity are reportedly suppressed, which can be relieved by melatonin by activating the p38 MAPK and PRKD1 signaling pathways to enhance osteoblast differentiation (Son et al., 2014; Zhou R. et al., 2020). In addition to promoting differentiation, melatonin can reverse apoptosis and damage osteoblasts and BMSCs (Qiu et al., 2019). Genetically speaking, melatonin can inhibit the development of adipogenesis of BMSC, which turns into bone development (Wang B. et al., 2019). The accumulation of lipids and LPL during adipogenic differentiation of hMSCs can be eliminated and reduced by melatonin. The logic is that, the receptor can induce degradation of β-catenin: peroxisome proliferator-activated receptor-γ (PPARγ), whose activation is blocked by melatonin. In addition, the levels of cAMP and ROS can be reduced by melatonin. In early adipocyte differentiation, cAMP triggers the activity of CCAAT/enhancer binding proteinβ, a critical inducer of PPARγ and C/EBPα activation, and the production of ROS further influences this activity. Melatonin can also downregulate the expression of lipogenic markers, such as C/EBPα, C/EBPβ, and p-ERK, thus enhancing the inhibition of the adipogenic differentiation of hMSCs (Rhee and Ahn, 2016). At a certain concentration, melatonin can partially inhibit the activation of the specific adipogenic transcription factor—— C/EBPβ (Alonso-Vale et al., 2009). Further research showed that melatonin significantly inhibited PPARγ expression but promoted the Runx2 expression in the early stages of adipogenesis and osteogenesis of hMSCs, with the synthesis of lipid droplets and the expression of adipocyte marker genes (LPL, adiponectin, aP2, and leptin) reduced to inhibit fat formation in human mesenchymal stem cells and enhance their osteogenic effects (Zhang et al., 2010). Zhou et al. found that melatonin can upregulate the osteogenic effects of BMSCs, but inhibit osteoclastogenesis via the MT2-mediated NF-κB pathway (Zhou Y. et al., 2020) (Figure 4).

It is well-established that melatonin inhibits osteolysis caused by osteoclasts. A study found that melatonin could inhibit the behavior of osteoclast differentiation, F-actin loop formation, and osteoclast absorption induced by RANKL *in vitro* in a concentration-dependent manner (Cutando et al., 2014). It has been reported that melatonin could inhibit the differentiation of RAW 264.7 cells into osteoclasts induced by light concentrations of RANKL and MCSF at the cellular level (Jarrar et al., 2020). Moreover, Kim et al. indicated that melatonin could inhibit osteoclast differentiation by downregulating the NF-κB pathway and reducing the induction of the NFATc1 transcription factor, though the phosphorylations of ERK, p38, and JNK were not affected. These results suggest that melatonin has an anti-osteoclast activity, which is not associated with MT1/MT2 receptors (Kim et al., 2017). Zhou et al. proposed that melatonin resists osteoclastogenesis in a dose-dependent manner, involving reactive oxygen species (ROS) mediation, but not the silent information modifier type 1 (SIRT1) independent pathway at pharmacological concentrations rather than physiological concentrations (Zhou et al., 2017), Recently, it has been found that in lung cancer and prostate cancer, melatonin can directly inhibit the production of RANKL in cancer cells by downregulating the p38 MAPK pathway, thereby preventing cancer-related osteoclast differentiation, bone resorption, and promoting the apoptosis of mature osteoclasts (Liu et al., 2021).

As an antioxidant, melatonin can scavenge free radicals and thereby affect bone metabolism. The appearance of oxidative stress will occur when the accumulation of free radicals exceeds the limit that the antioxidant system can withstand; in this particular situation, increased bone absorption and loss are accompanied by oxidative stress (Galano and Reiter, 2018). Melatonin pretreatment is reported to effectively reverse all changes in cytotoxicity and mitochondrial dysfunction caused by H2O2-induced oxidative stress in MG63 cells, which indicates that melatonin can maintain the oxidation homeostasis of H2O2-treated cells and the mitochondrial function. (She et al., 2014). Studies on diabetic rats show that melatonin administration can reduce biomarkers related to oxidative stress and has shown beneficial effects on short-term bone formation and healing in rats induced by the streptozotocin (STZ) effect (Kose et al., 2016). It is currently known that melatonin can reduce the ROS caused by autophagy injury through Sirt3/FOXO3a/Parkin signaling or by activating the AKT/mTOR pathway to link the pro-inflammatory effects of different tissues (Ma et al., 2018; Chen et al., 2021). It can activate the intracellular antioxidant Thioredoxin-1 (thereby suppressing the TXNIP/NLRP3 pathway) and inhibiting the impaired mitophagy (upregulating LC3B-II, PINK-1, Parkin, expression) (Ma et al., 2018).

#### Melatonin and implants

Given the great potential of melatonin in bone physiology, more emphasis has been placed on using melatonin in stomatology (Permuy et al., 2017). As previously mentioned, the key to the success rate of implants is osseointegration. A cell experiment found that melatonin could increase the proliferation and differentiation of MG63 osteoblasts cultured on a titanium sheet (Sola-Ruiz et al., 2017). Melatonin exerts a positive effect on promoting implant osseointegration; after 2 weeks of treatment, melatonin significantly increased the circumference, bone density, new bone formation, and inter-thread bone of the bone in direct contact with the treated implant compared to the control implant (Cutando et al., 2008; Gómez-Moreno et al., 2015). Although the role of MT on the bone-remodeling period has been established, the hematoma and inflammation period in the early stage of implant surgery also affect the success or failure of implant surgery, while melatonin has been reported to protect new bone formation in an inflammatory environment (Li X. et al., 2019). In the early stages after implant surgery, the inflammatory phase can be summarized as the formation of blood clots, ischemia and reperfusion injury, and the infiltration of inflammatory cells. In the meantime, neutrophils produce radicals, triggering a series of linkage effects and cell membrane damages through lipid peroxidation. Pineal melatonin is an important free-radical scavenger and antioxidant both physiologically and pharmacologically. Overwhelming evidence substantiates that the administration of melatonin may inhibit the effect of free-oxygen free-radicals and may regulate the activity of antioxidant enzymes in the process of fracture healing (Halıcı et al., 2010). During the hyperplasia phase, angiogenesis, differentiation, and infiltrating of osteoblast and fibroblast, collagen deposition, and granulation formation occur. A recent study showed that melatonin actively promotes vasculogenesis during bone defect repair in rabbits (Ramírez-Fernández et al., 2013). Finally, melatonin could improve the periodontal state and reduce gingivitis and periodontitis by reducing inflammatory cytokines, oxidative stress parameters, and periodontal damage (Cutando et al., 2014; Carpentieri et al., 2017). Indeed, melatonin has huge prospects for improving the prognosis of implants and the prevention and treatment of peri-implantitis.

#### Animal and cell experiments

So far, numerous animal models have confirmed the promotion of osteogenesis by the use of exogenous melatonin in dental implants (Cutando et al., 2008; Takechi et al., 2008; Calvo-Guirado et al., 2009; Calvo-Guirado et al., 2010; Guardia et al., 2011; Muñoz et al., 2012; Tresguerres et al., 2012; Calvo-Guirado et al., 2015; Salomó-Coll et al., 2016b; Dundar et al., 2016; Arora and Ivanovski, 2017; Palin et al., 2018; Sun et al., 2020; Xiao et al., 2020). Parameters used to evaluate the success of implant osseointegration include BIC, BV/TV, Tb.N, the mean connective density (Conn.D), Tb.Th, and so on. Most studies report that within a short period (ranging from 2 to 8 weeks) after implantation, melatonin significantly increases BIC, BV/TV, and Tb.N. After implant surgery, melatonin was systemically administered in some studies (Takechi et al., 2008; Palin et al., 2018; Sun et al., 2020; Xiao et al., 2020), while others applied melatonin topically (Cutando et al., 2008; Calvo-Guirado et al., 2009; Calvo-Guirado et al., 2010; Guardia et al., 2011; Muñoz et al., 2012; Tresguerres et al., 2012; Salomó-Coll et al., 2016b; Dundar et al., 2016) (Table 2), Palin et al. showed that the RUNX2, ALP, osteopontin (OP), and osteocalcin (OC) expressions were different in pinealectomized rats compared with the control group; after using melatonin, these expression levels could be restored to a certain extent (Palin et al., 2018), Sun et al. reported that the daily use of melatonin after implant surgery resulted in a difference in the bone morphological parameters, as the expression of the runt-related Runx2, OC, and osteoprotegerin genes were significantly upregulated and the RANKL gene was downregulated (Sun et al., 2020). Recently, some experimental studies have found that melatonin can inhibit osteolysis and osteoinhibition caused by titanium-wear particles through different signaling pathways (Zhang et al., 2020), which is helpful to further explore the pathway and mechanism of melatonin on the osseointegration of the implant surface.

**TABLE 2:** Summary of studies focusing on the use of melatonin in dental implant placements.

Author	Animal	Number	Postoperative observation time	Whether accompanied by other agents	Result and/or conclusions	Time
Guardia et al. (2011)	Beagle dogs	12	5w 8w	Alone	After 5 and 8 weeks of treatment, melatonin significantly increased inter-thread bone (*p* < 0.05) and new bone formation (*p* < 0.05) in 2 weeks compared with the control implant.	2011
Takechi et al. (2008)	Wistar rat	24	4w	FGF-2	These results strongly indicate that melatonin and FGF-2 have the potential to promote osseointegration.	2008
Cutando et al. (2008)	Beagle dogs	12	14d	Alone	Melatonin significantly increased the bone circumference (*p* < 0.0001), bone density (*p* < 0.0001), new bone formation (*p* < 0.0001) and inter-thread bone (*p* < 0.05) in direct contact with the treatment implant and control implants.	2008
Calvo-Guirado et al. (2009)	Beagle dogs	12	3m	discrete calcium deposit (DCD) surface	The combination of melatonin and porcine bone on the DCD implant shows more bone-to-implant contact.	2009
Tresguerres et al. (2012)	New Zealand rabbits	10	4w	Alone	Local application of melatonin during implant placement may induce more trabecular bone and higher trabecular area density at the implant contact.	2012
Muñoz et al. (2012)	Beagle dogs	12	2w 5w 8w	Growth hormone (GH)	GH and melatonin can synergistically promote the formation of new bone around titanium implants in the early stages of healing.	2012
Calvo-Guirado et al. (2015)	New Zealand rabbits	20	1w 4w	Alone	Topical application of melatonin increases the BIC value of titanium and zirconia implants at 1 week.	2015
Dundar et al. (2016)	New Zealand rabbits	6	4w	Alone	Within the limitations of this animal study, topical application of 5% melatonin or 10% vitamin D can improve bone formation around implants placed immediately after tooth extraction and help reduce CBL after 12 weeks of osseointegration.	2016
Salomó-Coll et al. (2016b)	American foxhound dogs	5	12w	Alone	Within the limitations of this animal study, the topical application of melatonin improved bone formation around immediate implants and reduced lingual bone and lingual peri-implant mucosa, after 12 weeks of osseointegration.	2016
Palin et al. (2018)	Wistar rat	18	60d	Alone	The loss of the pineal gland impairs the bone repair process during osseointegration, but daily supplementation of melatonin can restore this response.	2018
Sun et al. (2020)	SD rat	30	12w	Alone	Systemic melatonin can help improve the fixation of HA-coated implants by promoting Runx2, OC and OPG gene expression and inhibiting RANKL gene expression, even in osteopenic rats.	2020

#### Translation and application

An increasing amount of evidence from recently published studies suggests that melatonin can be used in implant coatings via sol-gel coatings (70M30T) as a release vehicle for MLT (Cerqueira et al., 2020). A composite adhesive hydrogel system (GelMA-DOPA@MT) was reported to achieve the sustained release of MT in the area of interest (Xiao et al., 2020), Accordingly, melatonin has huge potential for application in implants and related fields.

### The relationship between growth hormone levels and the osseointegration rate

#### Growth hormone

Growth hormone (GH) is a hormone secreted by pituitary somatotrophs in a pulsatile manner and acts on peripheral tissues, stimulating the liver and cartilage tissue to release a variety of bone-growth factors. Systemic GH treatment has been used in osteoporosis patients, reducing the risk of fractures and increasing bone turnover (Ohlsson and Vidal, 1998). The topical application of GH can also promote osteoblast differentiation and improve the mechanical properties and longitudinal growth of bones (Isaksson et al., 1982; Barnard et al., 1991). As for bone defects, the local application of GH can also increase the repair of biological materials (Guicheux et al., 1998).

For periodontal tissue, after 48 h of culture with GH, disparities in the expression of osteogenic markers, e.g., ALP, BMP, BSP, osteocalcin, and osteopontin, have been reported between periodontal cells and alveolar bone cells (Haase et al., 2003), which also lays the foundation for subsequent research (Figure 4).

Continuous administration of the systemic human growth hormone (hGH) to rabbits can reportedly promote the osseointegration of implants to a certain extent and improve the initial stability; nonetheless, long-term studies are lacking due to the rapid formation of antibodies (Stenport et al., 2001), In contrast, the topical use of the growth hormone has greater research prospects. Early research studies found that calcium phosphate-coated implants loaded with the growth hormone improved bone ingrowth and contact in grooves (Blom et al., 1998), Subsequently, further animal experimental studies corroborated that the topical use of the growth hormone could enhance the bone response around the implant, such as an increased periosteal response and corresponding cortical response and mineralization of osteoid (Tresguerres et al., 2002). W for biomechanics such as pull-out stress values, bone morphological parameters such as BIC and bone mineral density (BMD) are increased significantly (Martin-Monge et al., 2017). The local administration of the combination of melatonin and growth hormone could also synergistically enhance the formation of new bone around the titanium implant and increase BIC in the early stages of healing (Muñoz et al., 2012).

## Conclusion

Hormones represent high-efficiency information-transmitting chemical substances that undertake the indispensable tasks of coordinating metabolism, growth and development, and other physiological processes. They constitute an information exchange and cascade network, playing an essential role directly or indirectly in the various stages of bone growth, metabolism, damage and repair, and recovery after implant surgery. For example, in addition to the release of pituitary hormones and growth hormones, the pituitary also indirectly affects the release of sex hormones, adrenaline, and glucocorticoids. There is also a large amount of synergy and antagonism between hormones, such as the synergistic effect between leptin and insulin and the treatment of diabetes by melatonin osteogenic differentiation inhibition. More importantly, clinically, the lack of a single hormone is rarely observed and comprehensive and complex multiple endocrine disorders are more common. For example, elderly female patients may have both diabetes and estrogen deficiency. Hormone deficiency can reduce implant osseointegration by influencing the general systematic network. Correspondingly, exogenous hormones can be used as a treatment for diseases characterized by low bone mass and susceptibility to fractures or hormonal disorders as potential risk factors to increase bone mass and osseointegration success rates. Nonetheless, there are still research gaps in the experimental technology in this field. The influence of the complement system and antagonistic effect of hormones on the osseointegration of implants warrant further basic research. Clinically, patients tend to be cautious about hormone supplementation, emphasizing the need to improve our current understanding of hormone substitution and the synergy between hormones. In addition, although the femoral implant models of mice and rats represent the main animal models, no consensus has been reached on the optimal approach to assess the implantation methods so far. Currently available measurement methods are very different and cannot be used to conduct cross-data comparisons.

On the other hand, although animal experiments are relatively mature, there is still a lack of well-designed clinical follow-ups and mature epidemiological studies to positively reflect the relationship between human response hormones and osseointegration. More importantly, a significant emphasis should be placed on quantifying preoperative hormone levels. To improve the prognosis, the preoperative plan for at-risk patients can also be further improved with the help of endocrinologists. Moreover, the relative contraindications of implants should be redefined.

## References

[B1] Agas D. Amaroli A. Lacava G. Yanagawa T. Sabbieti M. G. (2020). Loss of p62 impairs bone turnover and inhibits PTH-induced osteogenesis. J. Cell. Physiol. 235, 7516–7529. 10.1002/jcp.29654 32100883

[B2] Aggarwal P. Zavras A. (2012). Parathyroid hormone and its effects on dental tissues. Oral Dis. 18, 48–54. 10.1111/j.1601-0825.2011.01850.x 21895887

[B3] Ajami E. Bell S. Liddell R. S. Davies J. E. (2016). Early bone anchorage to micro- and nano-topographically complex implant surfaces in hyperglycemia. Acta Biomater. 39, 169–179. 10.1016/j.actbio.2016.05.017 27181877

[B4] Al Zahrani S. Al Mutairi A. A. (2018). Stability and bone loss around submerged and non-submerged implants in diabetic and non-diabetic patients: A 7-year follow-up. Braz. Oral Res. 32, e57. 10.1590/1807-3107bor-2018.vol32.0057 29995063

[B5] Alonso-vale M. I. Peres S. B. Vernochet C. Farmer S. R. Lima F. B. (2009). Adipocyte differentiation is inhibited by melatonin through the regulation of C/EBPbeta transcriptional activity. J. Pineal Res. 47, 221–227. 10.1111/j.1600-079X.2009.00705.x 19663997

[B6] Annibali S. Pranno N. Cristalli M. P. LA Monaca G. Polimeni A. (2016). Survival analysis of implant in patients with diabetes mellitus: A systematic review. Implant Dent. 25, 663–674. 10.1097/ID.0000000000000478 27540845

[B7] Arora H. Ivanovski S. (2017). Melatonin as a pro-osteogenic agent in oral implantology: A systematic review of histomorphometric outcomes in animals and quality evaluation using ARRIVE guidelines. J. Periodontal Res. 52, 151–161. 10.1111/jre.12386 27098201

[B8] August M. Chung K. Chang Y. Glowacki J. (2001). Influence of estrogen status on endosseous implant osseointegration. J. Oral Maxillofac. Surg. 59, 1285–1289. 10.1053/joms.2001.27515 11688027

[B9] Barnard R. Ng K. W. Martin T. J. Waters M. J. (1991). Growth hormone (GH) receptors in clonal osteoblast-like cells mediate a mitogenic response to GH. Endocrinology 128, 1459–1464. 10.1210/endo-128-3-1459 1999164

[B10] Bassett J. H. Williams G. R. (2016). Role of thyroid hormones in skeletal development and bone maintenance. Endocr. Rev. 37, 135–187. 10.1210/er.2015-1106 26862888 PMC4823381

[B11] Blom E. J. Verheij J. G. DE Blieck-Hogervorst J. M. DI Silvio L. Klein C. P. (1998). Cortical bone ingrowth in growth hormone-loaded grooved implants with calcium phosphate coatings in goat femurs. Biomaterials 19, 263–270. 10.1016/s0142-9612(98)00205-1 9678875

[B12] Botolin S. Faugere M. C. Malluche H. Orth M. Meyer R. Mccabe L. R. (2005). Increased bone adiposity and peroxisomal proliferator-activated receptor-gamma2 expression in type I diabetic mice. Endocrinology 146, 3622–3631. 10.1210/en.2004-1677 15905321 PMC1242186

[B13] Calvo-Guirado J. L. Aguilar Salvatierra A. Gargallo-Albiol J. Delgado-Ruiz R. A. Maté Sanchez J. E. Satorres-Nieto M. (2015). Zirconia with laser-modified microgrooved surface vs. titanium implants covered with melatonin stimulates bone formation. Experimental study in tibia rabbits. Clin. Oral Implants Res. 26, 1421–1429. 10.1111/clr.12472 25155996

[B14] Calvo-Guirado J. L. Gómez-Moreno G. Barone A. Cutando A. Alcaraz-Baños M. Chiva F. (2009). Melatonin plus porcine bone on discrete calcium deposit implant surface stimulates osteointegration in dental implants. J. Pineal Res. 47, 164–172. 10.1111/j.1600-079X.2009.00696.x 19570131

[B15] Calvo-Guirado J. L. Gómez-Moreno G. López-Marí L. Guardia J. Marínez-González J. M. Barone A. (2010). Actions of melatonin mixed with collagenized porcine bone versus porcine bone only on osteointegration of dental implants. J. Pineal Res. 48, 194–203. 10.1111/j.1600-079x.2009.00743.x 20443224

[B16] Calvo-Guirado J. L. Mate-Sanchez J. Delgado-Ruiz R. Ramirez-Fernández M. P. Cutando-Soriano A. Peña M. (2011). Effects of growth hormone on initial bone formation around dental implants: A dog study. Clin. Oral Implants Res. 22, 587–593. 10.1111/j.1600-0501.2010.02007.x 21121954

[B17] Carpentieri A. R. Peralta Lopez M. E. Aguilar J. Solá V. M. (2017). Melatonin and periodontal tissues: Molecular and clinical perspectives. Pharmacol. Res. 125, 224–231. 10.1016/j.phrs.2017.09.003 28918172

[B18] Cauley J. A. (2015). Estrogen and bone health in men and women. Steroids 99, 11–15. 10.1016/j.steroids.2014.12.010 25555470

[B19] Cerqueira A. Romero-Gavilán F. Araújo-Gomes N. García-Arnáez I. Martinez-Ramos C. Ozturan S. (2020). A possible use of melatonin in the dental field: Protein adsorption and *in vitro* cell response on coated titanium. Mat. Sci. Eng. C Mat. Biol. Appl. 116, 111262. 10.1016/j.msec.2020.111262 32806297

[B20] Chen B. Lin T. Yang X. Li Y. Xie D. Cui H. (2016). Intermittent parathyroid hormone (1-34) application regulates cAMP-response element binding protein activity to promote the proliferation and osteogenic differentiation of bone mesenchymal stromal cells, via the cAMP/PKA signaling pathway. Exp. Ther. Med. 11, 2399–2406. 10.3892/etm.2016.3177 27284327 PMC4887846

[B21] Chen K. Zhu P. Chen W. Luo K. Shi X. J. Zhai W. (2021). Melatonin inhibits proliferation, migration, and invasion by inducing ROS-mediated apoptosis via suppression of the PI3K/Akt/mTOR signaling pathway in gallbladder cancer cells. Aging (Albany NY) 13, 22502–22515. 10.18632/aging.203561 34580235 PMC8507264

[B22] Chen M. Huang L. Shen X. Li M. Luo Z. Cai K. (2020). Construction of multilayered molecular reservoirs on a titanium alloy implant for combinational drug delivery to promote osseointegration in osteoporotic conditions. Acta Biomater. 105, 304–318. 10.1016/j.actbio.2020.01.029 31982586

[B23] Choukroun J. Khoury G. Khoury F. Russe P. Testori T. Komiyama Y. (2014). Two neglected biologic risk factors in bone grafting and implantology: High low-density lipoprotein cholesterol and low serum vitamin D. J. Oral Implantol. 40, 110–114. 10.1563/AAID-JOI-D-13-00062 24107195

[B24] Coelho P. G. Pippenger B. Tovar N. Koopmans S. J. Plana N. M. Graves D. T. (2018). Effect of obesity or metabolic syndrome and diabetes on osseointegration of dental implants in a miniature swine model: A pilot study. J. Oral Maxillofac. Surg. 76, 1677–1687. 10.1016/j.joms.2018.02.021 29572133 PMC6064394

[B25] Compston J. (2018a). Glucocorticoid-induced osteoporosis: An update. Endocrine 61, 7–16. 10.1007/s12020-018-1588-2 29691807 PMC5997116

[B26] Compston J. (2018b). Type 2 diabetes mellitus and bone. J. Intern. Med. 283, 140–153. 10.1111/joim.12725 29265670

[B27] Corsini M. S. Faraco F. N. Castro A. A. Onuma T. Sendyk W. R. Shibli J. A. (2008). Effect of systemic intermittent administration of human parathyroid hormone (rhPTH[1-34]) on the resistance to reverse torque in rabbit tibiae. J. Oral Implantol. 34, 298–302. 10.1563/1548-1336-34.6.298 19133483

[B28] Cutando A. Aneiros-Fernández J. López-Valverde A. Arias-Santiago S. Aneiros-Cachaza J. Reiter R. J. (2011). A new perspective in oral health: Potential importance and actions of melatonin receptors MT1, MT2, MT3, and RZR/ROR in the oral cavity. Arch. Oral Biol. 56, 944–950. 10.1016/j.archoralbio.2011.03.004 21459362

[B29] Cutando A. Gómez-Moreno G. Arana C. Muñoz F. Lopez-Peña M. Stephenson J. (2008). Melatonin stimulates osteointegration of dental implants. J. Pineal Res. 45, 174–179. 10.1111/j.1600-079X.2008.00573.x 18298460

[B30] Cutando A. López-Valverde A. DE Diego R. G. DE Vicente J. Reiter R. Fernández M. H. (2014). Effect of topical application of melatonin to the gingiva on salivary osteoprotegerin, RANKL and melatonin levels in patients with diabetes and periodontal disease. Odontology 102, 290–296. 10.1007/s10266-013-0122-5 23934086

[B31] Das S. Reddy M. A. Senapati P. Stapleton K. Lanting L. Wang M. (2018). Diabetes mellitus-induced long noncoding RNA Dnm3os regulates macrophage functions and inflammation via nuclear mechanisms. Arterioscler. Thromb. Vasc. Biol. 38, 1806–1820. 10.1161/ATVBAHA.117.310663 29930005 PMC6202204

[B32] Daugaard H. Elmengaard B. Andreassen T. T. Lamberg A. Bechtold J. E. Soballe K. (2012). Systemic intermittent parathyroid hormone treatment improves osseointegration of press-fit inserted implants in cancellous bone. Acta Orthop. 83, 411–419. 10.3109/17453674.2012.702388 22880714 PMC3427634

[B33] De molon R. S. Morais-Camilo J. A. Verzola M. H. Faeda R. S. Pepato M. T. Marcantonio E. (2013). Impact of diabetes mellitus and metabolic control on bone healing around osseointegrated implants: Removal torque and histomorphometric analysis in rats. Clin. Oral Implants Res. 24, 831–837. 10.1111/j.1600-0501.2012.02467.x 22509797

[B34] De oliveira P. Bonfante E. A. Bergamo E. T. P. DE Souza S. L. S. Riella L. Torroni A. (2020). Obesity/metabolic syndrome and diabetes mellitus on peri-implantitis. Trends Endocrinol. Metab. 31, 596–610. 10.1016/j.tem.2020.05.005 32591106

[B35] Ding Q. Sun P. Zhou H. Wan B. Yin J. Huang Y. (2018). Lack of endogenous parathyroid hormone delays fracture healing by inhibiting vascular endothelial growth factor-mediated angiogenesis. Int. J. Mol. Med. 42, 171–181. 10.3892/ijmm.2018.3614 29620150 PMC5979887

[B36] Duarte P. M. César Neto J. B. Gonçalves P. F. Sallum E. A. Nociti J F. (2003). Estrogen deficiency affects bone healing around titanium implants: A histometric study in rats. Implant Dent. 12, 340–346. 10.1097/01.id.0000099750.26582.4b 14752971

[B37] Dundar S. Yaman F. Saybak A. Ozupek M. F. Toy V. E. Gul M. (2016). Evaluation of effects of topical melatonin application on osseointegration of dental implant: An experimental study. J. Oral Implantol. 42, 386–389. 10.1563/aaid-joi-D-16-00048 27327091

[B38] Dvorak G. Fügl A. Watzek G. Tangl S. Pokorny P. Gruber R. (2012). Impact of dietary vitamin D on osseointegration in the ovariectomized rat. Clin. Oral Implants Res. 23, 1308–1313. 10.1111/j.1600-0501.2011.02346.x 22151621

[B39] Feitosa dda S. Bezerra Bde B. Ambrosano G. M. Nociti F. H. Casati M. Z. Sallum E. A. (2008). Thyroid hormones may influence cortical bone healing around titanium implants: A histometric study in rats. J. Periodontol. 79, 881–887. 10.1902/jop.2008.070466 18454667

[B40] Ferreira S. D. Silva G. L. Cortelli J. R. Costa J. E. Costa F. O. (2006). Prevalence and risk variables for peri-implant disease in Brazilian subjects. J. Clin. Periodontol. 33, 929–935. 10.1111/j.1600-051X.2006.01001.x 17092244

[B41] Fiorellini J. P. Sourvanos D. Crohin C. C. Crohin M. Chang J. J. Mattos M. (2020). Diabetic serum inhibits osteoblast adhesion to titanium surface through advanced glycation end products: An *in vitro* study. Int. J. Oral Maxillofac. Implants 35, 551–559. 10.11607/jomi.8114 32406653

[B42] Fretwurst T. Grunert S. Woelber J. P. Nelson K. Semper-Hogg W. (2016). Vitamin D deficiency in early implant failure: Two case reports. Int. J. Implant Dent. 2, 24. 10.1186/s40729-016-0056-0 27888492 PMC5124022

[B43] Galano A. Reiter R. J. (2018). Melatonin and its metabolites vs oxidative stress: From individual actions to collective protection. J. Pineal Res. 65, e12514. 10.1111/jpi.12514 29888508

[B44] Ge X. Li Z. Jing S. Wang Y. Li N. Lu J. (2020). Parathyroid hormone enhances the osteo/odontogenic differentiation of dental pulp stem cells via ERK and P38 MAPK pathways. J. Cell. Physiol. 235, 1209–1221. 10.1002/jcp.29034 31276209

[B45] Gomes-ferreira P. H. S. DE Oliveira D. Frigério P. B. DE Souza Batista F. R. Grandfield K. Okamoto R. (2020). Teriparatide improves microarchitectural characteristics of peri-implant bone in orchiectomized rats. Osteoporos. Int. 31, 1807–1815. 10.1007/s00198-020-05431-y 32383065

[B46] Gómez-moreno G. Aguilar-Salvatierra A. Boquete-Castro A. Guardia J. Piattelli A. Perrotti V. (2015). Outcomes of topical applications of melatonin in implant dentistry: A systematic review. Implant Dent. 24, 25–30. 10.1097/ID.0000000000000186 25621548

[B47] Grosso M. J. Courtland H. W. Yang X. Sutherland J. P. Stoner K. Nguyen J. (2015). Intermittent PTH administration and mechanical loading are anabolic for periprosthetic cancellous bone. J. Orthop. Res. 33, 163–173. 10.1002/jor.22748 25408434 PMC4776647

[B48] Guardia J. Gómez-Moreno G. Ferrera M. J. Cutando A. (2011). Evaluation of effects of topic melatonin on implant surface at 5 and 8 weeks in Beagle dogs. Clin. Implant Dent. Relat. Res. 13, 262–268. 10.1111/j.1708-8208.2009.00211.x 19681939

[B49] Guicheux J. Gauthier O. Aguado E. Heymann D. Pilet P. Couillaud S. (1998). Growth hormone-loaded macroporous calcium phosphate ceramic: *In vitro* biopharmaceutical characterization and preliminary *in vivo* study. J. Biomed. Mat. Res. 40, 560–566. 10.1002/(sici)1097-4636(19980615)40:4<560::aid-jbm7>3.0.co;2-d 9599032

[B50] Guido mangano F. Ghertasi Oskouei S. Paz A. Mangano N. Mangano C. (2018). Low serum vitamin D and early dental implant failure: Is there a connection? A retrospective clinical study on 1740 implants placed in 885 patients. J. Dent. Res. Dent. Clin. Dent. Prospects 12, 174–182. 10.15171/joddd.2018.027 30443302 PMC6231147

[B51] Haase H. R. Ivanovski S. Waters M. J. Bartold P. M. (2003). Growth hormone regulates osteogenic marker mRNA expression in human periodontal fibroblasts and alveolar bone-derived cells. J. Periodontal Res. 38, 366–374. 10.1034/j.1600-0765.2003.00655.x 12828652

[B52] Hadrowicz P. Hadrowicz J. Kozakiewicz M. Gesing A. (2017). Assessment of parathyroid hormone serum level as a predictor for bone condition around dental implants. Int. J. Oral Maxillofac. Implants 32, e207–e212. 10.11607/jomi.5686 28708916

[B53] Halıcı M. Öner M. Güney A. Canöz Ö. Narin F. HALıCı C. (2010). Melatonin promotes fracture healing in the rat model. Eklem Hast. Cerrahisi 21, 172–177. 21067500

[B54] Hasegawa H. Ozawa S. Hashimoto K. Takeichi T. Ogawa T. (2008). Type 2 diabetes impairs implant osseointegration capacity in rats. Int. J. Oral Maxillofac. Implants 23, 237–246. 18548919

[B55] Hassan N. Mccarville K. Morinaga K. Mengatto C. M. Langfelder P. Hokugo A. (2017). Titanium biomaterials with complex surfaces induced aberrant peripheral circadian rhythms in bone marrow mesenchymal stromal cells. PLoS One 12, e0183359. 10.1371/journal.pone.0183359 28817668 PMC5560683

[B56] He P. Zhang H. Li Y. Ren M. Xiang J. Zhang Z. (2020). 1α, 25-Dihydroxyvitamin D3-loaded hierarchical titanium scaffold enhanced early osseointegration. Mat. Sci. Eng. C Mat. Biol. Appl. 109, 110551. 10.1016/j.msec.2019.110551 32228967

[B57] Holick M. F. (2006). High prevalence of vitamin D inadequacy and implications for health. Mayo Clin. Proc. 81, 353–373. 10.4065/81.3.353 16529140

[B58] Holick M. F. (2004). Sunlight and vitamin D for bone health and prevention of autoimmune diseases, cancers, and cardiovascular disease. Am. J. Clin. Nutr. 80, 1678S–88s. 10.1093/ajcn/80.6.1678S 15585788

[B59] Holick M. F. (2002). Vitamin D: The underappreciated D-lightful hormone that is important for skeletal and cellular health. Curr. Opin. Endocrinol. Diabetes 9, 87–98. 10.1097/00060793-200202000-00011

[B60] Howe M. S. Keys W. Richards D. (2019). Long-term (10-year) dental implant survival: A systematic review and sensitivity meta-analysis. J. Dent. 84, 9–21. 10.1016/j.jdent.2019.03.008 30904559

[B61] Hu X. F. Wang L. Xiang G. Lei W. Feng Y. F. (2018). Angiogenesis impairment by the NADPH oxidase-triggered oxidative stress at the bone-implant interface: Critical mechanisms and therapeutic targets for implant failure under hyperglycemic conditions in diabetes. Acta Biomater. 73, 470–487. 10.1016/j.actbio.2018.04.008 29649637

[B62] Hua Y. Bi R. Li Z. Li Y. (2020). Resveratrol treatment promotes titanium implant osseointegration in diabetes mellitus rats. J. Orthop. Res. 38, 2113–2119. 10.1002/jor.24651 32141632

[B63] Huang X. Shu H. Ren C. Zhu J. (2022). SIRT3 improves bone regeneration and rescues diabetic fracture healing by regulating oxidative stress. Biochem. Biophys. Res. Commun. 604, 109–115. 10.1016/j.bbrc.2022.03.001 35303676

[B64] Isaksson O. G. Jansson J. O. Gause I. A. (1982). Growth hormone stimulates longitudinal bone growth directly. Science 216, 1237–1239. 10.1126/science.7079756 7079756

[B65] Jarrar H. Çeti̇n Altindal D. Gümüşdereli̇oğlu M. (2020). The inhibitory effect of melatonin on osteoclastogenesis of RAW 264.7 cells in low concentrations of RANKL and MCSF. Turk J. Biol. 44, 427–436. 10.3906/biy-2007-85 33402869 PMC7759193

[B66] Jiang L. Zhang W. Wei L. Zhou Q. Yang G. Qian N. (2018). Early effects of parathyroid hormone on vascularized bone regeneration and implant osseointegration in aged rats. Biomaterials 179, 15–28. 10.1016/j.biomaterials.2018.06.035 29960821

[B67] Jiang T. Xia C. Chen X. Hu Y. Wang Y. Wu J. (2019). Melatonin promotes the BMP9-induced osteogenic differentiation of mesenchymal stem cells by activating the AMPK/β-catenin signalling pathway. Stem Cell Res. Ther. 10, 408. 10.1186/s13287-019-1511-7 31864412 PMC6925474

[B68] Jiao H. Xiao E. Graves D. T. (2015). Diabetes and its effect on bone and fracture healing. Curr. Osteoporos. Rep. 13, 327–335. 10.1007/s11914-015-0286-8 26254939 PMC4692363

[B69] Kaback L. A. Soung Do Y. Naik A. Geneau G. Schwarz E. M. Rosier R. N. (2008). Teriparatide (1-34 human PTH) regulation of osterix during fracture repair. J. Cell. Biochem. 105, 219–226. 10.1002/jcb.21816 18494002 PMC3337675

[B70] Kassebaum N. J. Bernabé E. Dahiya M. Bhandari B. Murray C. J. Marcenes W. (2014). Global burden of severe tooth loss: A systematic review and meta-analysis. J. Dent. Res. 93, 20S–28s. 10.1177/0022034514537828 24947899 PMC4293725

[B71] Keller J. C. Stewart M. Roehm M. Schneider G. B. (2004). Osteoporosis-like bone conditions affect osseointegration of implants. Int. J. Oral Maxillofac. Implants 19, 687–694. 15508984

[B72] Kelly J. Lin A. Wang C. J. Park S. Nishimura I. (2009). Vitamin D and bone physiology: Demonstration of vitamin D deficiency in an implant osseointegration rat model. J. Prosthodont. 18, 473–478. 10.1111/j.1532-849X.2009.00446.x 19486459

[B73] Kim H. J. Kim H. J. Bae M. K. Kim Y. D. (2017). Suppression of osteoclastogenesis by melatonin: A melatonin receptor-independent action. Int. J. Mol. Sci. 18, E1142. 10.3390/ijms18061142 28587149 PMC5485966

[B74] Kitazawa R. Mori K. Yamaguchi A. Kondo T. Kitazawa S. (2008). Modulation of mouse RANKL gene expression by Runx2 and vitamin D3. J. Cell. Biochem. 105, 1289–1297. 10.1002/jcb.21929 18814144

[B75] Kose O. Arabaci T. Kara A. Yemenoglu H. Kermen E. Kizildag A. (2016). Effects of melatonin on oxidative stress index and alveolar bone loss in diabetic rats with periodontitis. J. Periodontol. 87, e82–90. 10.1902/jop.2016.150541 26832833

[B76] Kuchler U. Luvizuto E. R. Tangl S. Watzek G. Gruber R. (2011a). Short-term teriparatide delivery and osseointegration: A clinical feasibility study. J. Dent. Res. 90, 1001–1006. 10.1177/0022034511407920 21555773

[B77] Kuchler U. Spilka T. Baron K. Tangl S. Watzek G. Gruber R. (2011b). Intermittent parathyroid hormone fails to stimulate osseointegration in diabetic rats. Clin. Oral Implants Res. 22, 518–523. 10.1111/j.1600-0501.2010.02047.x 21251075

[B78] Kuroshima S. Kovacic B. L. Kozloff K. M. Mccauley L. K. Yamashita J. (2013). Intra-oral PTH administration promotes tooth extraction socket healing. J. Dent. Res. 92, 553–559. 10.1177/0022034513487558 23611925 PMC3654759

[B79] Kwiatek J. Jaroń A. Trybek G. (2021). Impact of the 25-hydroxycholecalciferol concentration and vitamin D deficiency treatment on changes in the bone level at the implant site during the process of osseointegration: A prospective, randomized, controlled clinical trial. J. Clin. Med. 10, 526. 10.3390/jcm10030526 33540512 PMC7867129

[B80] Kwon P. T. Rahman S. S. Kim D. M. Kopman J. A. Karimbux N. Y. Fiorellini J. P. (2005). Maintenance of osseointegration utilizing insulin therapy in a diabetic rat model. J. Periodontol. 76, 621–626. 10.1902/jop.2005.76.4.621 15857104

[B81] Lecka-czernik B. (2017). Diabetes, bone and glucose-lowering agents: Basic biology. Diabetologia 60, 1163–1169. 10.1007/s00125-017-4269-4 28434032 PMC5487688

[B82] Lee H. S. Heo H. A. Park S. H. Lee W. Pyo S. W. (2018). Influence of human parathyroid hormone during orthodontic tooth movement and relapse in the osteoporotic rat model: A preliminary study. Orthod. Craniofac. Res. 21, 125–131. 10.1111/ocr.12226 29671936

[B83] Li H. Zhou Q. Bai B. L. Weng S. J. Wu Z. Y. Xie Z. J. (2018). Effects of combined human parathyroid hormone (1-34) and menaquinone-4 treatment on the interface of hydroxyapatite-coated titanium implants in the femur of osteoporotic rats. J. Bone Min. Metab. 36, 691–699. 10.1007/s00774-017-0893-9 29280077

[B84] Li T. Jiang S. Lu C. Yang W. Yang Z. Hu W. (2019a). Melatonin: Another avenue for treating osteoporosis? J. Pineal Res. 66, e12548. 10.1111/jpi.12548 30597617

[B85] Li T. Wang H. Lv C. Huang L. Zhang C. Zhou C. (2021). Intermittent parathyroid hormone promotes cementogenesis via ephrinB2-EPHB4 forward signaling. J. Cell. Physiol. 236, 2070–2086. 10.1002/jcp.29994 32740946

[B86] Li X. Li Z. Wang J. Li Z. Cui H. Dai G. (2019b). Wnt4 signaling mediates protective effects of melatonin on new bone formation in an inflammatory environment. Faseb J. 33, 10126–10139. 10.1096/fj.201900093RR 31216173

[B87] Li X. Ma X. Y. Feng Y. F. Ma Z. S. Wang J. Ma T. C. (2015). Osseointegration of chitosan coated porous titanium alloy implant by reactive oxygen species-mediated activation of the PI3K/AKT pathway under diabetic conditions. Biomaterials 36, 44–54. 10.1016/j.biomaterials.2014.09.012 25308520

[B88] Li Y. Hu Z. Zhou C. Xu Y. Huang L. Wang X. (2017). Intermittent parathyroid hormone (PTH) promotes cementogenesis and alleviates the catabolic effects of mechanical strain in cementoblasts. BMC Cell Biol. 18, 19. 10.1186/s12860-017-0133-0 28427342 PMC5397739

[B89] Lian J. B. Stein G. S. (1993). The developmental stages of osteoblast growth and differentiation exhibit selective responses of genes to growth factors (TGF beta 1) and hormones (vitamin D and glucocorticoids). J. Oral Implantol. 19, 95–105. 8246305

[B90] Lima L. L. César Neto J. B. Cayana E. G. Nociti F. H. Sallum E. A. Casati M. Z. (2013). Parathyroid hormone (1-34) compensates the negative effect of smoking around implants. Clin. Oral Implants Res. 24, 1055–1059. 10.1111/j.1600-0501.2012.02502.x 22712894

[B91] Liu B. Gan X. Zhao Y. Yu H. Gao J. Yu H. (2019). Inhibition of HMGB1 promotes osseointegration under hyperglycemic condition through improvement of BMSC dysfunction. Oxid. Med. Cell. Longev., 1703709. 10.1155/2019/1703709 31929852 PMC6939424

[B92] Liu J. Huang F. He H. W. (2013). Melatonin effects on hard tissues: Bone and tooth. Int. J. Mol. Sci. 14, 10063–10074. 10.3390/ijms140510063 23665905 PMC3676828

[B93] Liu P. I. Chang A. C. Lai J. L. Lin T. H. Tsai C. H. Chen P. C. (2021). Melatonin interrupts osteoclast functioning and suppresses tumor-secreted RANKL expression: Implications for bone metastases. Oncogene 40, 1503–1515. 10.1038/s41388-020-01613-4 33452455

[B94] Liu W. Zhang S. Zhao D. Zou H. Sun N. Liang X. (2014). Vitamin D supplementation enhances the fixation of titanium implants in chronic kidney disease mice. PLoS One 9, e95689. 10.1371/journal.pone.0095689 24752599 PMC3994107

[B95] Liu Z. Zhou W. Tangl S. Liu S. Xu X. Rausch-Fan X. (2015). Potential mechanism for osseointegration of dental implants in Zucker diabetic fatty rats. Br. J. Oral Maxillofac. Surg. 53, 748–753. 10.1016/j.bjoms.2015.05.023 26093969

[B96] Luo C. Yang Q. Liu Y. Zhou S. Jiang J. Reiter R. J. (2019). The multiple protective roles and molecular mechanisms of melatonin and its precursor N-acetylserotonin in targeting brain injury and liver damage and in maintaining bone health. Free Radic. Biol. Med. 130, 215–233. 10.1016/j.freeradbiomed.2018.10.402 30315933

[B97] Lv Z. Muheremu A. Bai X. Zou X. Lin T. Chen B. (2020). PTH(1-34) activates the migration and adhesion of BMSCs through the rictor/mTORC2 pathway. Int. J. Mol. Med. 46, 2089–2101. 10.3892/ijmm.2020.4754 33125102 PMC7595657

[B98] Ma S. Chen J. Feng J. Zhang R. Fan M. Han D. (2018). Melatonin ameliorates the progression of atherosclerosis via mitophagy activation and NLRP3 inflammasome inhibition. Oxid. Med. Cell. Longev., 9286458. 10.1155/2018/9286458 30254716 PMC6142770

[B99] Ma X. Y. Feng Y. F. Ma Z. S. Li X. Wang J. Wang L. (2014). The promotion of osteointegration under diabetic conditions using chitosan/hydroxyapatite composite coating on porous titanium surfaces. Biomaterials 35, 7259–7270. 10.1016/j.biomaterials.2014.05.028 24912815

[B100] Maffezzoni F. Formenti A. M. (2018). Acromegaly and bone. Minerva Endocrinol. 43, 168–182. 10.23736/S0391-1977.17.02733-X 28880058

[B101] Mair B. Tangl S. Feierfeil J. Skiba D. Watzek G. Gruber R. (2009). Age-related efficacy of parathyroid hormone on osseointegration in the rat. Clin. Oral Implants Res. 20, 400–405. 10.1111/j.1600-0501.2008.01658.x 19298294

[B102] Mangano F. Mortellaro C. Mangano N. Mangano C. (2016). Is low serum vitamin D associated with early dental implant failure? A retrospective evaluation on 1625 implants placed in 822 patients. London, United Kingdom: Mediators of Inflammation, 531971. 10.1155/2016/5319718 PMC5055956 27738389

[B103] Martin-monge E. Tresguerres I. F. Clemente C. Tresguerres J. A. (2017). Local application of growth hormone to enhance osseointegration in osteoporotic bones: A morphometric and densitometric study. Int. J. Oral Maxillofac. Implants 32, 751–758. 10.11607/jomi.5320 28708907

[B104] Maus U. M. Lühmann M. Ohnsorge J. A. Andereya S. Schmidt H. Zombory G. (2013). Dihydrotestosterone improves the osseointegration of cobalt-chromium implants. Z. Orthop. Unf. 151, 25–30. 10.1055/s-0032-1328209 23423588

[B105] Mccracken M. Lemons J. E. Rahemtulla F. Prince C. W. Feldman D. (2000). Bone response to titanium alloy implants placed in diabetic rats. Int. J. Oral Maxillofac. Implants 15, 345–354. 10874799

[B106] Muñoz F. López-Peña M. Miño N. Gómez-Moreno G. Guardia J. Cutando A. (2012). Topical application of melatonin and growth hormone accelerates bone healing around dental implants in dogs. Clin. Implant Dent. Relat. Res. 14, 226–235. 10.1111/j.1708-8208.2009.00242.x 19793331

[B107] Naito Y. Jimbo R. Bryington M. S. Vandeweghe S. Chrcanovic B. R. Tovar N. (2014). The influence of 1α.25-dihydroxyvitamin d3 coating on implant osseointegration in the rabbit tibia. J. Oral Maxillofac. Res. 5, e3. 10.5037/jomr.2014.5303 PMC4219862 25386230

[B108] Needleman I. Garcia R. Gkranias N. Kirkwood K. L. Kocher T. Iorio A. D. (2018). Mean annual attachment, bone level, and tooth loss: A systematic review. J. Periodontol. 89 (1), S120–s139. 10.1002/JPER.17-0062 29926956

[B109] Ohlsson C. Vidal O. (1998). Effects of growth hormone and insulin-like growth factors on human osteoblasts. Eur. J. Clin. Invest. 28, 184–186. 10.1046/j.1365-2362.1998.00266.x 9568462

[B110] Olivares-navarrete R. Hyzy S. L. Boyan B. D. Schwartz Z. (2015). Regulation of osteoblast differentiation by acid-etched and/or grit-blasted titanium substrate topography is enhanced by 1, 25(OH)2D3 in a sex-dependent manner. Biomed. Res. Int., 365014. 10.1155/2015/365014 25945332 PMC4402479

[B111] Otawa M. Tanoue R. Kido H. Sawa Y. Yamashita J. (2015). Intermittent administration of parathyroid hormone ameliorates periapical lesions in mice. J. Endod. 41, 646–651. 10.1016/j.joen.2014.12.008 25649296 PMC4410053

[B112] Oteo-álvaro Á. Matas J. A. Alonso-Farto J. C. (2011). Teriparatide (rh [1-34] PTH) improved osteointegration of a hemiarthroplasty with signs of aseptic loosening. Orthopedics 34, e574–7. 10.3928/01477447-20110714-50 21902160

[B113] Palin L. P. Polo T. O. B. Batista F. R. S. Gomes-Ferreira P. H. S. Garcia Junior I. R. Rossi A. C. (2018). Daily melatonin administration improves osseointegration in pinealectomized rats. J. Appl. Oral Sci. 26, e20170470. 10.1590/1678-7757-2017-0470 29995145 PMC6025886

[B114] Pan J. Shirota T. Ohno K. Michi K. (2000). Effect of ovariectomy on bone remodeling adjacent to hydroxyapatite-coated implants in the tibia of mature rats. J. Oral Maxillofac. Surg. 58, 877–882. 10.1053/joms.2000.8212 10935587

[B115] Panda D. K. Miao D. Bolivar I. Li J. Huo R. Hendy G. N. (2004). Inactivation of the 25-hydroxyvitamin D 1alpha-hydroxylase and vitamin D receptor demonstrates independent and interdependent effects of calcium and vitamin D on skeletal and mineral homeostasis. J. Biol. Chem. 279, 16754–16766. 10.1074/jbc.M310271200 14739296

[B116] Pang X. Zhuang Y. Li Z. Jing S. Cai Q. Zhang F. (2020). Intermittent administration of parathyroid hormone enhances odonto/osteogenic differentiation of stem cells from the apical papilla via JNK and P38 MAPK pathways. Stem Cells Int. 2020, 5128128. 10.1155/2020/5128128 32148520 PMC7042551

[B117] Park J. Y. Heo H. A. Park S. Pyo S. W. (2020). Enhancement of peri-implant bone formation via parathyroid hormone administration in a rat model at risk for medication-related osteonecrosis of the jaw. J. Periodontal Implant Sci. 50, 121–131. 10.5051/jpis.2020.50.2.121 32395390 PMC7192826

[B118] Park K. H. Kang J. W. Lee E. M. Kim J. S. Rhee Y. H. Kim M. (2011). Melatonin promotes osteoblastic differentiation through the BMP/ERK/Wnt signaling pathways. J. Pineal Res. 51, 187–194. 10.1111/j.1600-079X.2011.00875.x 21470302

[B119] Pereira R. C. Salusky I. B. Bowen R. E. Freymiller E. G. Wesseling-Perry K. (2019). Vitamin D sterols increase FGF23 expression by stimulating osteoblast and osteocyte maturation in CKD bone. Bone 127, 626–634. 10.1016/j.bone.2019.07.026 31377240 PMC6715148

[B120] Permuy M. López-Peña M. González-Cantalapiedra A. Muñoz F. (2017). Melatonin: A review of its potential functions and effects on dental diseases. Int. J. Mol. Sci. 18, E865. 10.3390/ijms18040865 28422058 PMC5412446

[B121] Petsinis V. Kamperos G. Alexandridi F. Alexandridis K. (2017). The impact of glucocorticosteroids administered for systemic diseases on the osseointegration and survival of dental implants placed without bone grafting-A retrospective study in 31 patients. J. Craniomaxillofac. Surg. 45, 1197–1200. 10.1016/j.jcms.2017.05.023 28684069

[B122] Pezhman L. Tahrani A. Chimen M. (2021). Dysregulation of leukocyte trafficking in type 2 diabetes: Mechanisms and potential therapeutic avenues. Front. Cell Dev. Biol. 9, 624184. 10.3389/fcell.2021.624184 33692997 PMC7937619

[B123] Prati A. J. Casati M. Z. Ribeiro F. V. Cirano F. R. Pastore G. P. Pimentel S. P. (2013). Release of bone markers in immediately loaded and nonloaded dental implants: A randomized clinical trial. J. Dent. Res. 92, 161S–7s. 10.1177/0022034513504951 24158337 PMC3860065

[B124] Qiu X. Wang X. Qiu J. Zhu Y. Liang T. Gao B. (2019). Melatonin rescued reactive oxygen species-impaired osteogenesis of human bone marrow mesenchymal stem cells in the presence of tumor necrosis factor-alpha. Stem Cells Int., 6403967. 10.1155/2019/6403967 31582985 PMC6754961

[B125] Ramírez-fernández M. P. Calvo-Guirado J. L. DE-Val J. E. Delgado-Ruiz R. A. Negri B. Pardo-Zamora G. (2013). Melatonin promotes angiogenesis during repair of bone defects: A radiological and histomorphometric study in rabbit tibiae. Clin. Oral Investig. 17, 147–158. 10.1007/s00784-012-0684-6 22323056

[B126] Rapuri P. B. Gallagher J. C. Nawaz Z. (2007). Caffeine decreases vitamin D receptor protein expression and 1, 25(OH)2D3 stimulated alkaline phosphatase activity in human osteoblast cells. J. Steroid Biochem. Mol. Biol. 103, 368–371. 10.1016/j.jsbmb.2006.12.037 17223552

[B127] Rathinavelu S. Guidry-Elizondo C. Banu J. (2018). Molecular modulation of osteoblasts and osteoclasts in type 2 diabetes. J. Diabetes Res. 2018, 6354787. 10.1155/2018/6354787 30525054 PMC6247387

[B128] Reiter R. J. (1991). Melatonin: The chemical expression of darkness. Mol. Cell. Endocrinol. 79, C153–C158. 10.1016/0303-7207(91)90087-9 1936532

[B129] Rhee Y. H. Ahn J. C. (2016). Melatonin attenuated adipogenesis through reduction of the CCAAT/enhancer binding protein beta by regulating the glycogen synthase 3 beta in human mesenchymal stem cells. J. Physiol. Biochem. 72, 145–155. 10.1007/s13105-015-0463-3 26797706

[B130] Rorsman P. Braun M. (2013). Regulation of insulin secretion in human pancreatic islets. Annu. Rev. Physiol. 75, 155–179. 10.1146/annurev-physiol-030212-183754 22974438

[B131] Rybaczek T. Tangl S. Dobsak T. Gruber R. Kuchler U. (2015). The effect of parathyroid hormone on osseointegration in insulin-treated diabetic rats. Implant Dent. 24, 392–396. 10.1097/ID.0000000000000288 26126148

[B132] Saito N. Mikami R. Mizutani K. Takeda K. Kominato H. Kido D. (2022). Impaired dental implant osseointegration in rat with streptozotocin-induced diabetes. J. Periodontal Res. 57, 412–424. 10.1111/jre.12972 35037248

[B133] Salomó-Coll O. DE Maté-Sánchez J. E. V. Ramírez-Fernandez M. P. Hernández-Alfaro F. Gargallo-Albiol J. Calvo-Guirado J. L. (2018). Osseoinductive elements around immediate implants for better osteointegration: A pilot study in foxhound dogs. Clin. Oral Implants Res. 29, 1061–1069. 10.1111/clr.12809 26923181

[B134] Salomó-Coll O. Maté-Sánchez DE Val J. E. Ramírez-Fernandez M. P. Hernández-Alfaro F. Gargallo-Albiol J. Calvo-Guirado J. L. (2016a). Topical applications of vitamin D on implant surface for bone-to-implant contact enhance: A pilot study in dogs part II. Clin. Oral Implants Res. 27, 896–903. 10.1111/clr.12707 26419393

[B135] Salomó-Coll O. Maté-Sánchez DE Val J. E. Ramírez-Fernández M. P. Satorres-Nieto M. Gargallo-Albiol J. Calvo-Guirado J. L. (2016b). Osseoinductive elements for promoting osseointegration around immediate implants: A pilot study in the foxhound dog. Clin. Oral Implants Res. 27, e167–e175. 10.1111/clr.12596 25833366

[B136] Schlegel K. A. Prechtl C. Möst T. Seidl C. Lutz R. VON Wilmowsky C. (2013). Osseointegration of SLActive implants in diabetic pigs. Clin. Oral Implants Res. 24, 128–134. 10.1111/j.1600-0501.2011.02380.x 22111960

[B137] Sghaireen M. G. Alduraywish A. A. Srivastava K. C. Shrivastava D. Patil S. R. AL Habib S. (2020). Comparative evaluation of dental implant failure among healthy and well-controlled diabetic patients-A 3-year retrospective study. Int. J. Environ. Res. Public Health 17, E5253. 10.3390/ijerph17145253 32708165 PMC7400304

[B138] Sharan K. Lewis K. Furukawa T. Yadav V. K. (2017). Regulation of bone mass through pineal-derived melatonin-MT2 receptor pathway. J. Pineal Res. 63, e12423. 10.1111/jpi.12423 PMC5575491 28512916

[B139] She F. Wang W. Wang Y. Tang P. Wei J. Chen H. (2014). Melatonin protects MG63 osteoblast-like cells from hydrogen peroxide-induced cytotoxicity by maintaining mitochondrial function. Mol. Med. Rep. 9, 493–498. 10.3892/mmr.2013.1832 24297096

[B140] Sheyn D. Cohn Yakubovich D. Kallai I. Su S. Da X. Pelled G. (2013). PTH promotes allograft integration in a calvarial bone defect. Mol. Pharm. 10, 4462–4471. 10.1021/mp400292p 24131143 PMC3902084

[B141] Shirota T. Tashiro M. Ohno K. Yamaguchi A. (2003). Effect of intermittent parathyroid hormone (1-34) treatment on the bone response after placement of titanium implants into the tibia of ovariectomized rats. J. Oral Maxillofac. Surg. 61, 471–480. 10.1053/joms.2003.50093 12684966

[B142] Shyng Y. C. Devlin H. Ou K. L. (2006). Bone formation around immediately placed oral implants in diabetic rats. Int. J. Prosthodont. 19, 513–514. 17323732

[B143] Silva M. A. Vasconcelos D. F. Marques M. R. Barros S. P. (2016). Parathyroid hormone intermittent administration promotes delay on rat incisor eruption. Arch. Oral Biol. 69, 102–108. 10.1016/j.archoralbio.2016.05.017 27285944

[B144] Sola-ruiz M. F. Perez-Martinez C. Labaig-Rueda C. Carda C. Martín DE Llano J. J. (2017). Behavior of human osteoblast cells cultured on titanium discs in relation to surface roughness and presence of melatonin. Int. J. Mol. Sci. 18, E823. 10.3390/ijms18040823 28406458 PMC5412407

[B145] Son J. H. Cho Y. C. Sung I. Y. Kim I. R. Park B. S. Kim Y. D. (2014). Melatonin promotes osteoblast differentiation and mineralization of MC3T3-E1 cells under hypoxic conditions through activation of PKD/p38 pathways. J. Pineal Res. 57, 385–392. 10.1111/jpi.12177 25250639

[B146] Stenport V. F. Olsson B. Morberg P. Törnell J. Johansson C. B. (2001). Systemically administered human growth hormone improves initial implant stability: An experimental study in the rabbit. Clin. Implant Dent. Relat. Res. 3, 135–141. 10.1111/j.1708-8208.2001.tb00133.x 11799703

[B147] Stratton I. M. Adler A. I. Neil H. A. Matthews D. R. Manley S. E. Cull C. A. (2000). Association of glycaemia with macrovascular and microvascular complications of type 2 diabetes (UKPDS 35): Prospective observational study. Bmj 321, 405–412. 10.1136/bmj.321.7258.405 10938048 PMC27454

[B148] Sun R. Liang C. Sun Y. Xu Y. Geng W. Li J. (2021). Effects of metformin on the osteogenesis of alveolar BMSCs from diabetic patients and implant osseointegration in rats. Oral Dis. 28, 1170–1180. 10.1111/odi.13808 33606350

[B149] Sun T. Li J. Xing H. L. Tao Z. S. Yang M. (2020). Melatonin improves the osseointegration of hydroxyapatite-coated titanium implants in senile female rats. Z. Gerontol. Geriatr. 53, 770–777. 10.1007/s00391-019-01640-1 31654128

[B150] Sundar G. Sridharan S. Sundaram R. R. Prabhu S. Rao R. Rudresh V. (2019). Impact of well-controlled type 2 diabetes mellitus on implant stability and bone biomarkers. Int. J. Oral Maxillofac. Implants 34, 1441–1449. 10.11607/jomi.7547 31184637

[B151] Takechi M. Tatehara S. Satomura K. Fujisawa K. Nagayama M. (2008). Effect of FGF-2 and melatonin on implant bone healing: A histomorphometric study. J. Mat. Sci. Mat. Med. 19, 2949–2952. 10.1007/s10856-008-3416-3 18360797

[B152] Tam C. S. Heersche J. N. Murray T. M. Parsons J. A. (1982). Parathyroid hormone stimulates the bone apposition rate independently of its resorptive action: Differential effects of intermittent and continuous administration. Endocrinology 110, 506–512. 10.1210/endo-110-2-506 7056211

[B153] Tang C. Li X. Wang F. Cui X. Zhu Y. (2015). Effect of local application of insulin like growth factor-1 gelatin sponge complex on osseointegration around implant in osteoporosis rats. Zhonghua Kou Qiang Yi Xue Za Zhi 50, 418–422. 26564746

[B154] Tang J. Yan D. Chen L. Shen Z. Wang B. Weng S. (2020). Enhancement of local bone formation on titanium implants in osteoporotic rats by biomimetic multilayered structures containing parathyroid hormone (PTH)-related protein. Biomed. Mat. 15, 045011. 10.1088/1748-605X/ab7b3d 32109901

[B155] Tao Z. S. Zhou W. S. Qiang Z. Tu K. K. Huang Z. L. Xu H. M. (2016). Intermittent administration of human parathyroid hormone (1-34) increases fixation of strontium-doped hydroxyapatite coating titanium implants via electrochemical deposition in ovariectomized rat femur. J. Biomater. Appl. 30, 952–960. 10.1177/0885328215610898 26482573

[B156] Terheyden H. Lang N. P. Bierbaum S. Stadlinger B. (2012). Osseointegration--communication of cells. Clin. Oral Implants Res. 23, 1127–1135. 10.1111/j.1600-0501.2011.02327.x 22092345

[B157] Tresguerres I. F. Clemente C. Blanco L. Khraisat A. Tamimi F. Tresguerres J. A. (2012). Effects of local melatonin application on implant osseointegration. Clin. Implant Dent. Relat. Res. 14, 395–399. 10.1111/j.1708-8208.2010.00271.x 20455901

[B158] Tresguerres I. F. Clemente C. Donado M. Gómez-Pellico L. Blanco L. Alobera M. A. (2002). Local administration of growth hormone enhances periimplant bone reaction in an osteoporotic rabbit model. Clin. Oral Implants Res. 13, 631–636. 10.1034/j.1600-0501.2002.130609.x 12519338

[B159] Ursomanno B. L. Cohen R. E. Levine M. J. Yerke L. M. (2020). The effect of hypothyroidism on bone loss at dental implants. J. Oral Implantol. 47, 131–134. 10.1563/aaid-joi-D-19-00350 32662836

[B160] Valderrama P. Jung R. E. Thoma D. S. Jones A. A. Cochran D. L. (2010). Evaluation of parathyroid hormone bound to a synthetic matrix for guided bone regeneration around dental implants: A histomorphometric study in dogs. J. Periodontol. 81, 737–747. 10.1902/jop.2010.090562 20429653

[B161] Wang B. Song Y. Wang F. Li D. Zhang H. Ma A. (2011). Effects of local infiltration of insulin around titanium implants in diabetic rats. Br. J. Oral Maxillofac. Surg. 49, 225–229. 10.1016/j.bjoms.2010.03.006 20400213

[B162] Wang B. Wen H. Smith W. Hao D. He B. Kong L. (2019a). Regulation effects of melatonin on bone marrow mesenchymal stem cell differentiation. J. Cell. Physiol. 234, 1008–1015. 10.1002/jcp.27090 30145787

[B163] Wang J. Meng F. Song W. Jin J. Ma Q. Fei D. (2018). Nanostructured titanium regulates osseointegration via influencing macrophage polarization in the osteogenic environment. Int. J. Nanomedicine 13, 4029–4043. 10.2147/IJN.S163956 30022825 PMC6045901

[B164] Wang L. Hu X. Ma X. Ma Z. Zhang Y. Lu Y. (2016). Promotion of osteointegration under diabetic conditions by tantalum coating-based surface modification on 3-dimensional printed porous titanium implants. Colloids Surf. B Biointerfaces 148, 440–452. 10.1016/j.colsurfb.2016.09.018 27648775

[B165] Wang Q. Nie L. Zhao P. Zhou X. Ding Y. Chen Q. (2021). Diabetes fuels periodontal lesions via GLUT1-driven macrophage inflammaging. Int. J. Oral Sci. 13, 11. 10.1038/s41368-021-00116-6 33762572 PMC7990943

[B166] Wang X. Qi F. Xing H. Zhang X. Lu C. Zheng J. (2019b). Uniform-sized insulin-loaded PLGA microspheres for improved early-stage peri-implant bone regeneration. Drug Deliv. 26, 1178–1190. 10.1080/10717544.2019.1682719 31738084 PMC6882491

[B167] Wojda S. J. Donahue S. W. (2018). Parathyroid hormone for bone regeneration. J. Orthop. Res. 36, 2586–2594. 10.1002/jor.24075 29926970

[B168] Wu Y. Y. Yu T. Yang X. Y. Li F. Ma L. Yang Y. (2013). Vitamin D3 and insulin combined treatment promotes titanium implant osseointegration in diabetes mellitus rats. Bone 52, 1–8. 10.1016/j.bone.2012.09.005 22985888

[B169] Xiao L. Lin J. Chen R. Huang Y. Liu Y. Bai J. (2020). Sustained release of melatonin from GelMA liposomes reduced osteoblast apoptosis and improved implant osseointegration in osteoporosis. Oxid. Med. Cell. Longev. 2020, 6797154. 10.1155/2020/6797154 32566094 PMC7275204

[B170] Xiong Y. Zhang Y. Guo Y. Yuan Y. Guo Q. Gong P. (2017). 1α, 25-Dihydroxyvitamin D(3) increases implant osseointegration in diabetic mice partly through FoxO1 inactivation in osteoblasts. Biochem. Biophys. Res. Commun. 494, 626–633. 10.1016/j.bbrc.2017.10.024 29080745

[B171] Xu Y. Lv C. Zhang J. Li Y. Li T. Zhang C. (2019). Intermittent parathyroid hormone promotes cementogenesis in a PKA- and ERK1/2-dependent manner. J. Periodontol. 90, 1002–1013. 10.1002/JPER.18-0639 31026057

[B172] Yamazaki S. Masaki C. Nodai T. Tsuka S. Tamura A. Mukaibo T. (2020). The effects of hyperglycaemia on peri-implant tissues after osseointegration. J. Prosthodont. Res. 64, 217–223. 10.1016/j.jpor.2019.07.007 31852608

[B173] Yary T. Virtanen J. K. Ruusunen A. Tuomainen T. P. Voutilainen S. (2016). Serum zinc and risk of type 2 diabetes incidence in men: The kuopio ischaemic heart disease risk factor study. J. Trace Elem. Med. Biol. 33, 120–124. 10.1016/j.jtemb.2015.11.001 26653753

[B174] Yin G. Liu H. Li J. Liu Y. Liu X. Luo E. (2019). Adenoviral delivery of adiponectin ameliorates osteogenesis around implants in ovariectomized rats. J. Gene Med. 21, e3069. 10.1002/jgm.3069 30609197

[B175] Yu X. Wang L. Jiang X. Rowe D. Wei M. (2012). Biomimetic CaP coating incorporated with parathyroid hormone improves the osseointegration of titanium implant. J. Mat. Sci. Mat. Med. 23, 2177–2186. 10.1007/s10856-012-4682-7 22639151

[B176] Yuan Y. Jiang Y. Wang B. Guo Y. Gong P. Xiang L. (2020). Deficiency of calcitonin gene-related peptide affects macrophage polarization in osseointegration. Front. Physiol. 11, 733. 10.3389/fphys.2020.00733 32848807 PMC7412000

[B177] Zhang K. Wang M. Li Y. Li C. Tang S. Qu X. (2019). The PERK-EIF2α-ATF4 signaling branch regulates osteoblast differentiation and proliferation by PTH. Am. J. Physiol. Endocrinol. Metab. 316, E590–E604. 10.1152/ajpendo.00371.2018 30668150

[B178] Zhang L. Su P. Xu C. Chen C. Liang A. DU K. (2010). Melatonin inhibits adipogenesis and enhances osteogenesis of human mesenchymal stem cells by suppressing PPARγ expression and enhancing Runx2 expression. J. Pineal Res. 49, 364–372. 10.1111/j.1600-079X.2010.00803.x 20738756

[B179] Zhang Y. Zhu X. Wang G. Chen L. Yang H. He F. (2020). Melatonin rescues the Ti particle-impaired osteogenic potential of bone marrow mesenchymal stem cells via the SIRT1/SOD2 signaling pathway. Calcif. Tissue Int. 107, 474–488. 10.1007/s00223-020-00741-z 32767062

[B180] Zhou C. Li Y. Wang X. Shui X. Hu J. (2012). 1, 25Dihydroxy vitamin D(3) improves titanium implant osseointegration in osteoporotic rats. Oral Surg. Oral Med. Oral Pathol. Oral Radiol. 114, S174–S178. 10.1016/j.oooo.2011.09.030 23063395

[B181] Zhou L. Chen X. Yan J. Li M. Liu T. Zhu C. (2017). Melatonin at pharmacological concentrations suppresses osteoclastogenesis via the attenuation of intracellular ROS. Osteoporos. Int. 28, 3325–3337. 10.1007/s00198-017-4127-8 28956094 PMC9841502

[B182] Zhou R. Ma Y. Tao Z. Qiu S. Gong Z. Tao L. (2020a). Melatonin inhibits glucose-induced apoptosis in osteoblastic cell line through PERK-eIF2α-ATF4 pathway. Front. Pharmacol. 11, 602307. 10.3389/fphar.2020.602307 33390989 PMC7772242

[B183] Zhou Y. Wang C. Si J. Wang B. Zhang D. Ding D. (2020b). Melatonin up-regulates bone marrow mesenchymal stem cells osteogenic action but suppresses their mediated osteoclastogenesis via MT(2) -inactivated NF-κB pathway. Br. J. Pharmacol. 177, 2106–2122. 10.1111/bph.14972 31900938 PMC7161576

[B184] Zhu G. Ma B. Dong P. Shang J. Gu X. Zi Y. (2020). Melatonin promotes osteoblastic differentiation and regulates PDGF/AKT signaling pathway. Cell Biol. Int. 44, 402–411. 10.1002/cbin.11240 31535749

